# Development of LncRNA Biomarkers in Extracellular Vesicle of Amniotic Fluid Associated with Antenatal Hydronephrosis

**DOI:** 10.3390/biomedicines13030668

**Published:** 2025-03-08

**Authors:** Ying Fu, Qiaoshu Liu, Ruojin Yao, Yimei Fu, Lei Dai, Wenyan Jian, Weishe Zhang, Jingzhi Li

**Affiliations:** 1 Department of Obstetrics, Xiangya Hospital, Central South University, Changsha 410008, China; fuying99@csu.edu.cn (Y.F.); 15973198441@163.com (Q.L.); ruojinyao@aliyun.com (R.Y.); fuyimei@csu.edu.cn (Y.F.); austindai@hotmail.com (L.D.); 18890353280@163.com (W.J.); zhangweishe@yeah.net (W.Z.); 2 NHC Key Laboratory of Cancer Proteomics & State Local Joint Engineering Laboratory for Anticancer Drugs, Xiangya Hospital, Central South University, Changsha 410008, China; 3 Hunan Engineering Research Center of Early Life Development and Disease Prevention, Changsha 410008, China

**Keywords:** antenatal hydronephrosis, amniotic fluid, small extracellular vesicles, lncRNAs

## Abstract

**Background**: Antenatal hydronephrosis (ANH) is the most common congenital renal and urinary tract anomaly, and parenchymal damage and renal fibrosis due to pathological hydronephrosis are the main causes of end-stage renal disease in children and chronic kidney disease in adults. At present, there is no validated biomarker for ANH, and diagnostic criteria other than prenatal ultrasonography (US) assessment are lacking. Therefore, we assessed to determine if biomarkers extracted from amniotic fluid small extracellular vesicles (sEVs) might be used as ANH diagnosis. **Methods**: With congenital ureteropelvic junction obstruction (UPJO) as the ultimate diagnosis, 10 pregnant women with Grade III-IV ANH and 10 normal pregnant women were recruited. The sEVs were extracted from amniotic fluid supernatant of all samples. Transcriptomic sequencing of sEVs in the discovery cohort identified the differential expression profiles for ANH. The known differentially expressed lncRNAs (DE-lncRNAs) were assessed by qRT–PCR in the validation cohort. **Results**: We explored the global RNA expression in sEVs from amniotic fluid. The differential expression profiles of both mRNAs and lncRNAs were related to fetal kidney development. Six known DE-lncRNAs were identified for ANH, and three of those with high expression were verified in more ANH samples. In particular, the upregulated LINC02863 and its target genes were associated with renal development and morphogenesis. The four predicted novel lncRNAs in high expression were also related to mesenchymal morphogenesis and the STAT3 signaling pathway and may play roles in ANH. **Conclusions**: We identified differentially expressed RNAs of all species in the sEVs from amniotic fluid, and the validated known DE-lncRNAs might serve as promising diagnostic biomarkers for ANH.

## 1. Introduction

Antenatal hydronephrosis (ANH) is one of the most common congenital anomalies affecting the kidney and urinary tract (CAKUT), with a series of clinical manifestations characterized by the separation of the renal pelvis collecting system or by dilatation of the calyces or ureters [1]. Pathological hydronephrosis occurs in 0.3–0.6% of newborns, and anomalies and pathological conditions after birth include ureteropelvic junction obstruction (UPJO), posterior urethral valves (PUV), ureteral obstruction, etc. [2,3]. Approximately 15–20% of children with ANH need surgery, especially when both kidneys are blocked, which leads to the rapid deterioration of renal function [4]. Therefore, early detection and intervention are important means to prevent the loss of renal function caused by ANH.

The management of ANH has been controversial for several decades. The distinction between transient hydronephrosis and UPJO remains one of the most controversial challenges [5,6]. Prenatal ultrasonography (US) and magnetic resonance imaging are the most commonly used methods for detecting and diagnosing ANH during pregnancy, and ultrasonic parameters such as amniotic fluid volume, renal cortical thinning, and fetal renal parenchyma structure, especially fetal anteroposterior renal pelvic diameter (APD/APRPD), are considered predictive indicators of postnatal renal function [7]. However, APD, the most common method for determining ANH grade, is a single measurement standard and has poor accuracy in predicting patient prognosis. There is an urgent need to develop accurate and practical biomarkers for the early diagnosis of ANH.

It is well known that urine is a promising source of biomarkers for kidney-related diseases [8,9]. Amniotic fluid (AF), which is mainly derived from fetal urine, is also an unexploited but potentially very valuable resource for assessing fetal renal status and development. In the clinic, the risk of amniocentesis is much lower than that of renal pelvis aspiration (RPA). AF is collected via amniocentesis during the second trimester, and AF cells are routinely used to detect fetal complications, such as fetal genetic abnormalities, fetal infections, and encephalopathies [10]. AF supernatant contains various potential biomarkers, including lipids, DNA, RNA, proteins, and small extracellular vesicles (sEVs) [11,12,13,14]. Moreover, an increasing number of studies have shown that sEV-derived long RNA species, including messenger RNA (mRNA), circular RNA (circRNA), and long noncoding RNA (lncRNA), have functional and clinical implications [15,16]. Xie et al. proposed that HSA-miR-300 and HSA-miR-299-5p in AF sEVs might serve as biomarkers for the diagnosis of congenital obstructive kidney disease and renal fibrosis [11]. LncRNAs in sEVs were reported to serve as diagnostic and prognostic biomarkers for various diseases [17], including kidney disease. LncRNAs, transcripts greater than 200 nucleotides in length, are expressed not only at the cellular or tissue level but also during specific phases of development [18]. They also modulate progression and drug resistance in kidney-related tumors [19]. So far, studies examining the association between sEV lncRNAs and ANH are rare.

In this study, we aimed to perform comprehensive AF-derived sEV profiling of RNA AF-derived sEVs and further elucidate the potential mechanism of ANH. We extracted sEVs from the discarded supernatant of routine amniocenteses from ultimate UPJO fetuses with severe ANH during the second trimester and performed a transcriptomic analysis of AF supernatant sEVs. The expression profiles and biological functions of both the mRNAs and known lncRNAs were investigated, and novel lncRNAs were also predicted and explored. Functional annotations of the cis-/trans-target genes and enrichment analyses of the differentially expressed lncRNAs (DE-lncRNAs) were performed to identify the significant biological functions and signaling pathways associated with embryonic kidney development. Quantitative real-time polymerase chain reaction (qRT–PCR) was used to verify the hub DE-lncRNAs, and the three upregulated lncRNAs were identified as candidate biomarkers for ANH diagnosis. This study may provide new insights into the pathogenesis of ANH and lay the foundation for the use of lncRNAs as diagnostic biomarkers in the future.

## 2. Materials and Methods

### 2.1. Patients

In total, 10 pregnant women with Grade III-IV ANH and 10 normal pregnant women in the second trimester of pregnancy were recruited from May 2009 to May 2022 at the Xiangya Hospital Obstetrics Department. To rule out fetal genetic problems, prenatal testing was compulsory for all of them. Routine amniocentesis was also conducted during mid-pregnancy. The basic information, detailed US, and postnatal diagnosis are summarized in Table 1 and Supplementary Tables S1 and S2. Sonographers specializing in obstetrics evaluated the quantitative values of APD, calyceal/ureteral dilation, and renal pelvis splitting using the Society for Fetal Urology (SFU) 5-point numerical grading system [2], and the data were collected from medical records. Postnatal diagnosis was obtained through follow-up. Informed consent was signed by all participants, and the ethics committee of Xiangya Hospital approved this study. All methods were performed in accordance with the relevant guidelines and regulations.

### 2.2. Sample Handling

The acquired AF samples were centrifuged at 3000× *g* for 15 min at 4 °C, after which the AF supernatant was retained. To minimize contamination of the platelets, the supernatant was transferred to new tubes and centrifuged at 3000× *g* for 15 min at 4 °C. The supernatant was filtered through a 0.22 μm filter and stored at −80 °C until isolation and identification.

### 2.3. Isolation of sEVs by Size-Exclusion Chromatography (SEC)

Ten-milliliter AF supernatant samples were thawed at 4 °C overnight. Then, the thawed samples were concentrated in a 50 mL 100 kDa filtration tube (1:100) and centrifuged at 4000× *g* for 15 min. One milliliter of condensed AF supernatant was loaded into a Sepharose-based CL-2B column (Echo9103A-10 mL; ECHO BIOTECH, Beijing China), which was prewashed with more than 20 mL of sterile PBS in advance. When no liquid was observed flowing out of the bottom of the columns after the addition of a complete sample into the columns, PBS was used to elute the sEVs and other fractions. Each 500 µL of effluent represented one fraction. A 100 kDa ultrafiltration tube was used to further purify the 4 to 7 fractions collected. We collected the enriched sEVs into a tube and centrifuged them for 15 min at 4000× *g* to enrich them [20].

### 2.4. Nanoparticle Tracking Analysis (NTA)

The vesicle-enriched suspension was stabilized at concentrations between 1 × 10^7^/mL and 1 × 10^9^/mL. The particles were examined by a ZetaView PMX 110 instrument (Particle Metrix, Meerbusch, Germany) equipped with a 405 nm laser, which determines the size and quantity of the particles isolated. An analysis of particle movement was conducted using NTA software via a video captured with a frame rate of 30 frames/second over a duration of 60 s (ZetaView 8.02.28) [21,22].

### 2.5. Transmission Electron Microscopy (TEM)

We incubated 20 µL of enriched sEVs on a copper mesh for 10 min at room temperature. After washing with sterile distilled water, the sEV-enriched fraction was stained with uranyl oxalate solution for 1 min and then allowed to dry under an incandescent lamp for 2 min. Images were observed and taken under a transmission electron microscope (JEOL-JEM1400, Tokyo, Japan) [23].

### 2.6. Western Blot (WB) Analysis

The proteins of the enriched sEVs were denatured in 5× sodium dodecyl sulfonate (SDS) loading buffer and subjected to Western blot analysis (10% SDS–polyacrylamide gel electrophoresis; 50 μg protein/lane). The following antibodies were used for detection: CD24 (67627-1-IG; proteintech, Wuhan, China), TSG101 (sc-13,611; Santa Cruz, CA, USA), and calnexin (10,427–2-AP; Promega, Madison, WI, USA). The results were visualized on a Tanon 4600 automatic chemiluminescence image analysis system (Tanon, Shanghai, China).

### 2.7. RNA Isolation and Sequencing

Total RNA was extracted and purified from sEV-enriched fractions using an miRNeasy^®^ Mini Kit (Qiagen, cat. No. 217,004) according to the manufacturer’s instructions. Agarose gels (1.5%) were used to monitor the degradation and contamination of RNA, especially DNA. Using a NanoDrop 2000 spectrophotometer (Thermo Fisher Scientific, Wilmington, DE, USA), we measured the concentration and purity of the RNA. RNA integrity was analyzed using an Agilent Bioanalyzer 2100 system (Agilent Technologies, Santa Clara, CA, USA) and an RNA Nano 6000 assay kit.

In the experimental group, 6 samples, including severe ANH (*n* = 3) and normal (*n* = 3) sEV samples, were subjected to library preparation and sequencing. For mRNAs and lncRNAs, a total of 5 ng of RNA per sample was analyzed with a Ribo-ZeroTM Magnetic Kit (Epicenter, Madison, WI, USA) to remove rRNA. The sequencing libraries were generated on an Ovation RNA-Seq system (NuGEN, Redwood City, CA, USA) following the manufacturer’s instructions, and index codes were used to attribute the sequences to the samples. The RNA samples were ligated with barcodes containing unique adaptor sequences to allow pooling of the samples. The pooled DNA library was then eluted and processed with a NovaSeq 6000 platform for cluster generation and sequencing. The quality of the libraries was assessed using a Qubit fluorometer (Thermo Fisher Scientific, Waltham, MA, USA).

For each sample, no less than 65 M clean reads were generated and the Q30 percentage was no less than 87.25% for sequencing. The raw reads were filtered by fastQC. The transcriptome was assembled using StringTie based on the reads mapped to the GRCh38 human genome by using HISAT2 [24]. The assembled transcripts were annotated using the gffcompare program. StringTie (1.3.1) [25] was used to calculate FPKMs of both lncRNAs and coding genes in each sample. Differential expression analysis of the two groups was performed using the edgeR R package [26].

### 2.8. Known lncRNA Identification

The analysis of known lncRNAs was based on the high-confidence set of the lncipedia database version 5.2. The lncipedia database integrates lncRNAdb, the Broad Institute (Human Body Map lincRNAs), Ensembl (release 92), GENCODE, RefSeq, NONCODE, and several other databases and lncRNAs in the literature. The transcripts of the lncRNA genes were included in the high-confidence dataset of lncipedia version 5.2.

### 2.9. Quantitative Real-Time PCR (qRT–PCR) Analysis

In the validation cohort, RNA samples from ANH patients (*n* = 7) and normal individuals (*n* = 7) were reverse transcribed into complementary DNA (cDNA) using HiScript Reverse Transcriptase (Vazyme; cat #R101-01) according to the manufacturer’s instructions. The cDNA was PCR-processed using ChamQ Universal SYBR qPCR Master Mix (Vazyme; cat #Q711-02). Real-time quantitative PCR (qPCR) was performed on a Roche LightCycler 480 II instrument. The qPCR was performed with the following procedure: 37 cycles at 94 °C for 15 s and 62 °C for 7 min. The primers used in this study are listed in Supplementary Table S3. Ct values for each sample were determined and normalized to that of β-actin and miR-16-5p, respectively. The 2^−ΔΔC(t)^ equation was used to calculate the relative expression data.

### 2.10. Prediction of Novel lncRNAs

The discovery of novel lncRNAs was divided into two stages: basic screening and potential coding ability screening. First, transcripts with a fragment per kilobase of exon model per million mapped fragments (FPKM) ≥ 0.1, a transcript length ≥ 200 bp, and a total number of exons ≥ 2 were selected. Following basic screening, we obtained transcript sequence information, and after removing transcripts with potential coding ability, the remaining novel predicted lncRNAs were identified. Since lncRNAs do not encode proteins, the transcripts were excluded because of the coding potential of the candidate lncRNAs. The most widely used coding potential analysis methods were also combined for further screening, mainly including Coding Potential Calculator (CPC), Coding Non-Coding Index (CNCI), Coding Potential Assessment Tool (CPAT), and Pfam protein structural domain analysis. CPC was used primarily to compare transcripts with known protein databases. Coding-noncoding transcripts were distinguished by adjacent nucleotide triplet features to predict noncoding transcripts (score < 0). CPAT analysis determined the coding and noncoding ability of transcripts by building logistic regression models and calculating Fickett and hexamer scores based on ORF length and ORF coverage. The Pfam database provides a comprehensive classification system for protein structural domain annotation; transcripts with a specific protein structural domain that was thought to have coding ability were excluded.

### 2.11. Prediction of Novel lncRNA Target Genes

Based on the mode of interaction of the lncRNAs and their target genes, two prediction methods were used. Cis-target genes are involved since lncRNAs regulate the expression of neighboring genes, which is mainly predicted based on the spatial relationship between lncRNAs and genes, and neighboring genes within a 100 kb region around the genomic location of these lncRNAs are potential target genes. The trans-target genes and the interactions between lncRNAs and mRNAs were determined based on their complementary pairing. LncTar was used for calculating the free energy of the pairing and normalized site of the complementary sequence between lncRNAs and mRNAs, and those below the normalized free energy threshold were considered lncRNA target genes.

### 2.12. Differential Expression Analysis of Novel lncRNAs

StringTie software was used to normalize the expression of novel lncRNAs. StringTie software simultaneously assembled and quantified the transcript sequence, yielding more quantitative and accurate results. The FPKM value was also used as the output to measure the expression levels of the novel lncRNAs.

### 2.13. Statistical Analysis

We used plyr and reshape2 to sort and restructure the sequence data. The plots were generated using corrplot (0.92), ggbiplot (0.55), ggvenn (0.1.9), ggplot2 (3.3.6), pheatmap (1.0.12), circlize (0.4.15), or ComplexHeatmap (1.10.2). Statistical tests were performed using R 3.5.1 (http://www.r-project.org, accessed on 8 February 2023); *p* < 0.05 and |log2FC| > 1 indicate differentially expressed RNAs. Wilcoxon rank-sum tests were used for group comparisons. The qPCR data were analyzed using GraphPad Prism 8.0. The unpaired *t* test was used for comparisons of the relative expression levels of candidate lncRNAs via qRT–PCR. The data in all the figures are expressed as the mean ± standard deviation (SD). A significance level of *p* < 0.05 was used. The *q*-value is the adjusted *p* value found using the optimized false discovery rate (FDR) method.

## 3. Results

### 3.1. Characterization and Properties of sEVs Isolated from AF

**Figure 1:**
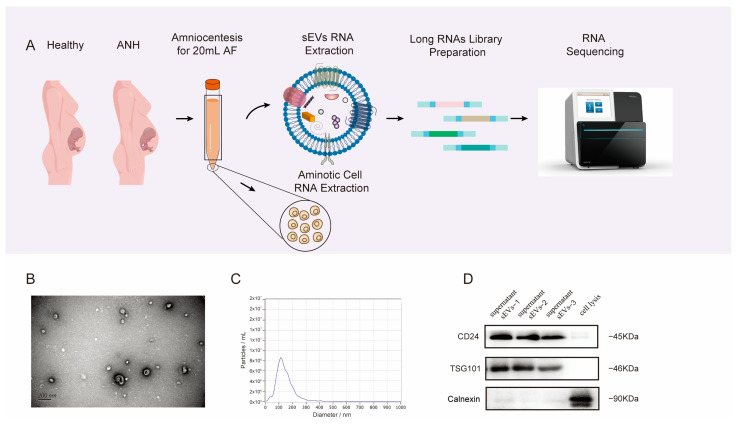
Schematic workflow showing the RNA-seq analysis of sEVs isolated from AF and its characterization. (**A**) Workflow of long RNA-seq of AF. (**B**) Electron microscopy image of sEVs isolated from the AF. (**C**) Size distribution measurements of sEVs isolated from the AF. (**D**) Western blot analysis of typical markers of sEVs isolated from AF.

The workflow of this study is shown in Figure 1A. The sEVs were extracted from the AF-derived supernatant of ANH and normal samples by using SEC methods and then characterized according to the MISEV2023 guidelines [27]. TEM images showed that the isolated sEVs had intact membrane structures and morphologies (Figure 1B). In a nanoparticle tracking analysis (NTA), the mean diameters of the isolated fractions were measured and ranged from 75 to 200 nm for most particles (Figure 1C). Subsequently, the expression of sEV-positive and sEV-negative markers was detected in our isolated sEV-enriched fractions by Western blotting. It was reported that CD24 can be used as a marker of sEVs that were isolated from the urine of healthy individuals or newborn infants and from the AF of pregnant women [28]. The results showed that the positive markers CD24 and TSG101 were strongly expressed and that the negative marker calnexin was absent among the sEVs (Figure 1D).

### 3.2. Differentially Expressed sEV-Derived mRNAs in ANH

**Figure 2:**
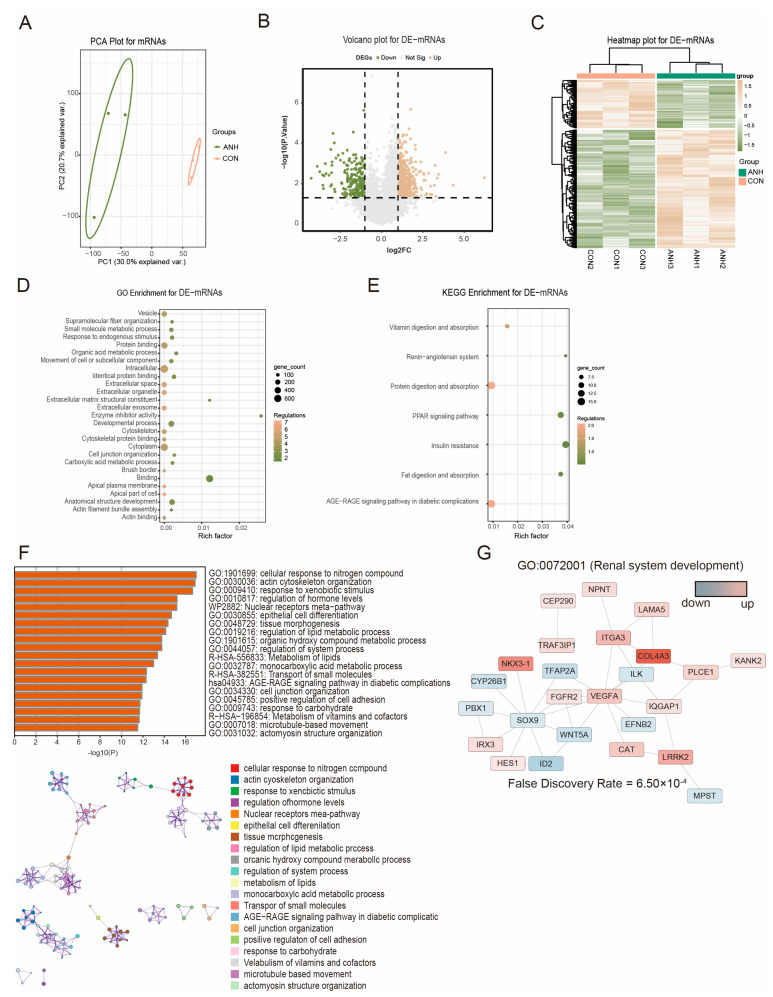
DE-mRNA profiles of sEVs isolated from the AF of the ANH and control groups. (**A**) Principal component analysis (PCA) of sEV-derived mRNAs of AF isolated from the ANH and control groups. (**B**) Volcano plots showing the statistical analysis of DE-mRNAs between the ANH and control groups. (**C**) Heatmap showing the DE-mRNAs between the ANH and control groups. (**D**) Bubble plot showing the numbers of DE-mRNAs in each GO enrichment. (**E**) Bubble plot showing the numbers of DE-mRNAs in each KEGG pathway. (**F**) The top 20 Metascape-enriched KEGG pathways and PPI network analysis of the DE-mRNAs. (**G**) PPI network analysis of mRNAs related to renal system development according to GO:0072001. ANH: ANH samples; CON: control samples.

Due to the particularity and rarity of severe ANH samples, mRNA and lncRNA expression in a total of 6 samples, including 3 severe ANH cases (SFU III-IV) and 3 normal cases (Table 1), was assessed by sequencing. First, the differentially expressed sEV-derived mRNAs between the ANH and control samples were analyzed. Principal component analysis (PCA) showed the separation of the two groups (Figure 2A). A *p* < 0.05 and |log2FC| > 1 were considered as the filtering criteria for differentially expressed mRNAs. As shown in the volcano plot (Figure 2B) and heatmap (Figure 2C), a total of 836 DE-mRNAs were identified, of which 421 were upregulated and 415 were downregulated. GO and KEGG analyses were performed for the DE-mRNAs. The enrichment results showed that these genes were involved mainly in vesicle, intracellular, and cytoplasm functions (Figure 2D) and were enriched in nutrient digestion and absorption (Figure 2E). Enrichment analysis was also performed using Metascape. The main processes included epithelial morphogenesis, tissue differentiation, cell motility, and oxidative metabolism of macromolecules such as monosaccharides and fatty acids (Figure 2F). We subsequently focused on renal-related pathways. The protein–protein interaction (PPI) network associated with renal system development (GO:0072001) was enriched, and the expression of key transcription factors involved in developmental regulation, such as SOX9, IRX3, NKX3-1, and other regulators, was upregulated (Figure 2G).

### 3.3. Differential and Functional Analysis of Known sEV-Derived lncRNAs in ANH

**Figure 3:**
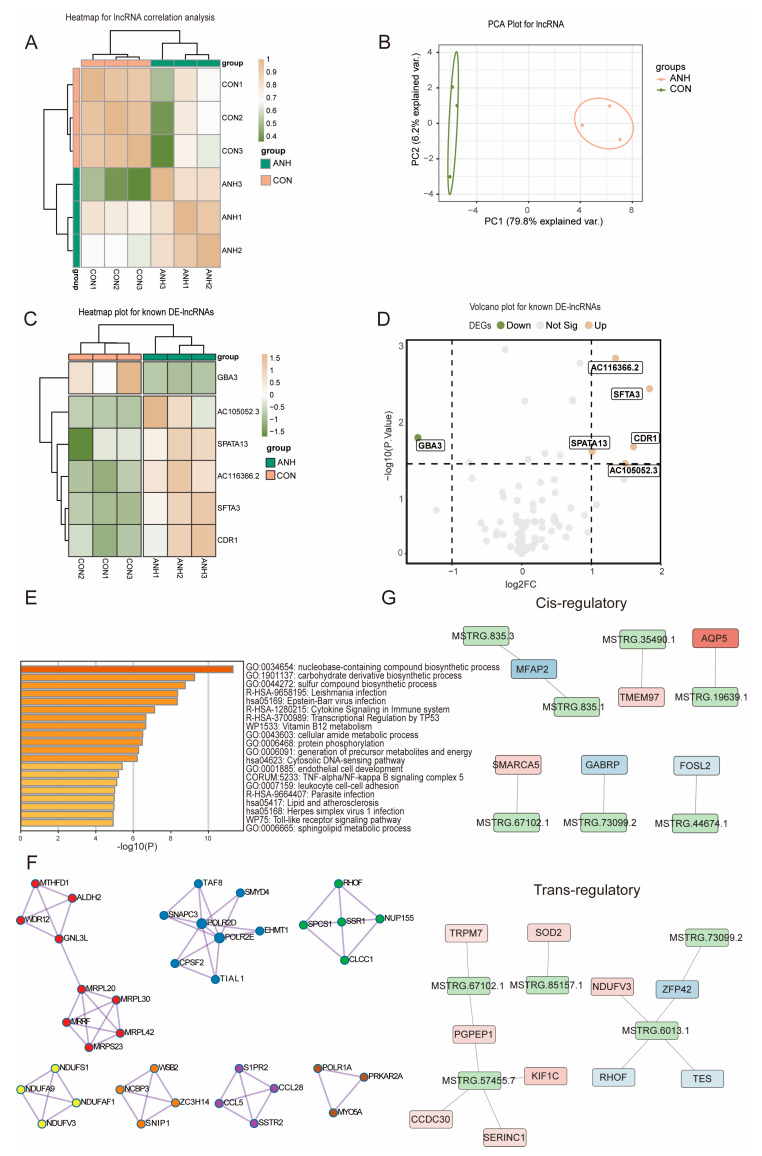
Expression and network analysis of the known DE-lncRNAs with their corresponding targeted genes in the ANH and control groups. (**A**) Pearson correlation analysis of lncRNAs between the ANH and control groups. (**B**) Principal component analysis (PCA) of lncRNAs between the ANH and control groups. (**C**) Heatmap showing the known DE-lncRNAs between the ANH and control groups. (**D**) Volcano plots showing the statistical analysis of the six known DE-lncRNAs identified in the ANH and control groups. (**E**) The top 20 Metascape-enriched KEGG pathways of the DE-lncRNA-targeted genes. (**F**) PPI network analysis of DE-lncRNA-targeted genes. (**G**) Cis- and trans-regulatory network analysis of DE-genes and DE-lncRNAs screened by Cytoscape (version 3.8.0). ANH: ANH samples; CON: control samples.

Bioinformatics analysis of the identified lncRNAs was also performed. Pearson correlation analysis (Figure 3A) and PCA (Figure 3B) indicated relatively good stability between the samples, that the samples in each group were highly identical, and that the differences between groups were obvious. There were 17,780 annotated initial genes, the majority of which were protein-coding genes (99.3%). The identified known lncRNAs included 69 pseudogenes, 21 antisense lncRNAs, 17 lincRNAs (long intergenic noncoding RNAs), 3 sense lncRNAs, and 1 intronic lncRNA. As shown by the heatmap (Figure 3C) and volcano plot (Figure 3D), the DEG analysis identified 5 upregulated and 1 downregulated DE-lncRNA. The detailed information of the 6 DE-lncRNAs is listed in Table 2.

To explore the potential function of the identified DE-lncRNAs, cis- or trans-target genes were also predicted. Cis-target gene prediction was based on genomic position relationships, and trans-target gene prediction was based on complementary sequences. A total of 70 cis-based (Cis) and 442 trans-target genes (Trans) were identified as known DE-lncRNAs. Both GO and KEGG analyses were employed for the target genes. The top 20 enriched pathways were related to several biosynthetic processes, infections, and metabolic regulations (Figure 3E). A PPI network (Figure 3F) and cis- and trans-regulatory networks were also constructed (Figure 3G).

### 3.4. Functional Analysis and Validation of the Known DE-lncRNAs in ANH

**Figure 4:**
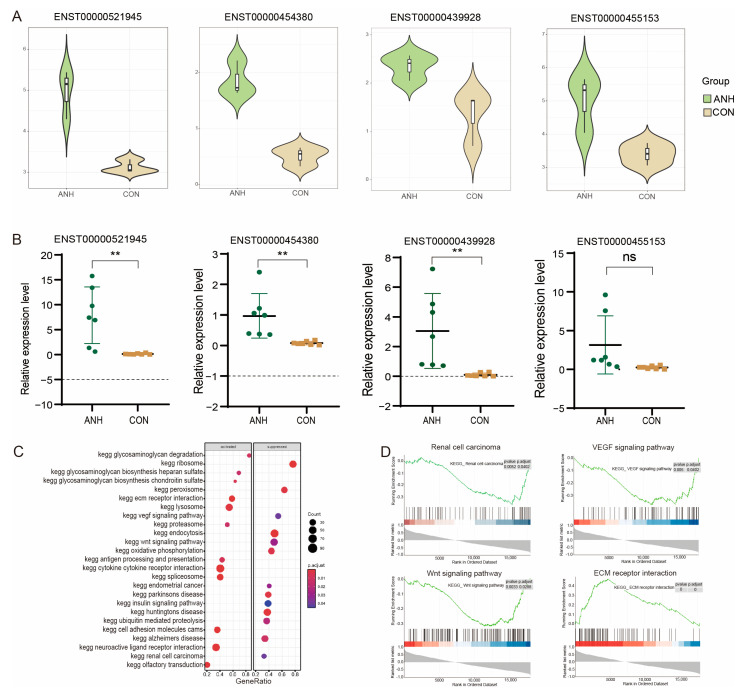
Expression analysis and validation of the selected known DE-lncRNAs. (**A**) Violin plot showing the expression analysis of four selected known DE-lncRNAs. (**B**) The qRT–PCR analysis of four selected sEV lncRNAs in severe ANH and control samples. (**C**) KEGG analysis of signaling pathways related to ENST00000454380. (**D**) GSEA of signaling pathways related to ENST00000454380. ANH: ANH samples; CON: control samples. The data are presented as the means ± SDs. ** *p* < 0.01; ns indicates no significance.

To further explore the potential of DE-lncRNAs as biomarkers for ANH, four of the upregulated DE-lncRNAs (Figure 4A), namely, ENST00000521945, ENST00000454380, ENST00000439928, and ENST00000455153, were selected for validation by qRT–PCR analysis. A total of 14 samples (severe ANH = 7, control = 7) were collected for the validation cohort (Supplementary Table S1). The results indicated that the expression levels of three of the four selected lncRNAs (ENST00000521945, ENST00000454380, and ENST00000439928) were significantly upregulated in the AF-derived sEVs of severe ANHs compared with those of the controls (Figure 4B and Figure S1).

Furthermore, the target genes were predicted for ENST00000454380, and seven possible cis-regulatory genes (MIR3936, SLC22A5, C5orf56, AC116366.2, Y_RNA, AC116366.1, and IRF1) were identified. These genes were located in the upper and lower reaches of the ENST00000454380 100 kb region. A total of 303 trans-regulatory target genes were also identified through the complementary sequences between the lncRNAs and mRNAs (Supplementary Table S4). The paired box 8 (PAX8) gene, a characteristic developmental marker gene in the kidney [29], was predicted to interact with ENST00000454380. Pearson correlation analysis was performed on the ENST00000454380 expression with its target genes in the expression dataset, after which KEGG enrichment analysis was conducted. The most significant pathways affected were involved in intercellular signaling (Figure 4C), such as interactions between cytokines and their receptors (*q* = 3.455 × 10^−7^), extracellular matrix receptor interactions (*q* = 1.163 × 10^−7^), neuroactive factor receptor ligand interactions (*q* = 1.269 × 10^−5^), cell adhesion molecules (*q* = 2.310 × 10^−5^), and endocytosis (*q* = 1.090 × 10^−5^). GSEA also revealed that this gene was negatively associated with genitourinary tract tumor-related genes, such as those related to endometrial cancer (*q* = 2.297 × 10^−2^) and renal cell carcinoma (*q* = 3.089 × 10^−2^). Pathways involved in tissue morphogenesis and cell proliferation, such as the Wnt pathway (*q* = 2.216 × 10^−2^), insulin pathway (*q* = 3.454 × 10^−2^), and epidermal growth factor pathway (*q* = 3.087 × 10^−2^), were also enriched. These results suggest that ENST00000454380, which was significantly highly expressed in ANH, may be related to cytokine interactions, development, proliferation, and tissue formation (Figure 4D) and may play critical roles in ANH.

### 3.5. Prediction and Functional Analysis of the Novel DE-lncRNAs in ANH

**Figure 5:**
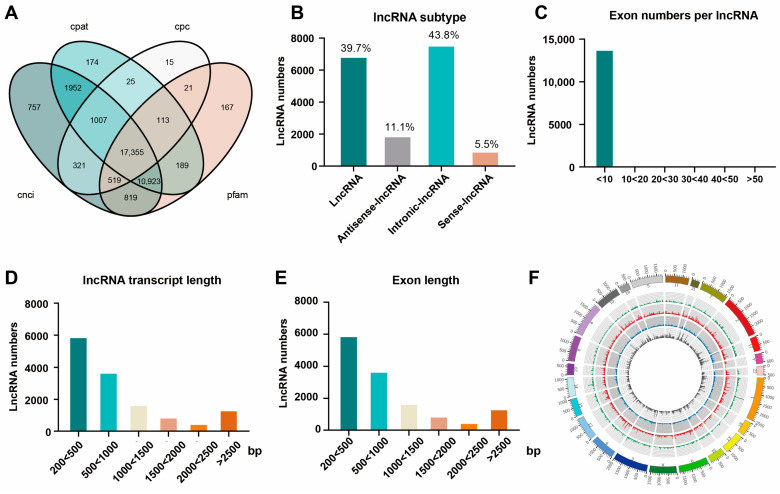
Prediction and identification of novel sEV-derived lncRNA profiles in the ANH and control groups. (**A**) Venn diagram showing the novel sEV-derived lncRNA predictions generated by using four methods (CNCI, CPC, Pfam, and CPAT). (**B**) Bar chart showing the distribution of novel lncRNA numbers based on genomic location isoforms. (**C**) Bar chart showing the distribution of exons in the novel lncRNAs. (**D**) Bar chart showing the distribution of the transcript lengths of the novel lncRNAs. (**E**) Bar chart showing the distribution of exon lengths of novel lncRNAs. (**F**) A loop diagram showing the chromosomal distribution of novel lncRNAs. The chromosomes, the sense lncRNAs (green), the lincRNAs (red), the antisense lncRNAs (blue), and the intronic lncRNAs (gray) are arranged from the outside to the inside.

A combined analysis of four methods (CPC, CNCI, CPAT, and Pfam) was used for novel lncRNAs, and Venn diagrams showing 17355 potential noncoding transcripts were obtained (Figure 5A). Based on their genomic locations, four different types were classified, including 39.7% of lincRNAs, 11.1% of antisense lncRNAs, 43.8% of intronic lncRNAs, and 5.5% of sense lncRNA transcripts (Figure 5B). A series of expression property analyses were performed. The average number of exons in the novel lncRNAs was approximately 2.89, and the majority of them (96.5%) had fewer than 10 exons (Figure 5C). The average transcript length of the novel lncRNAs was 1179 bp, and that of MSTRG.34386 was the longest (64001 bp) (Figure 5D). Furthermore, the average length of exons was approximately 408 bp, 50.2% of which were less than 200 bp in length (Figure 5E). The chromosome distributions of the novel lncRNAs are also shown in Figure 5F. The most abundant lncRNAs were distributed on chromosome 1 (*n* = 3885), followed by chromosome 2 (*n* = 3392). The fewest lncRNAs were also found in the mitochondrial genome (*n* = 2).

**Figure 6:**
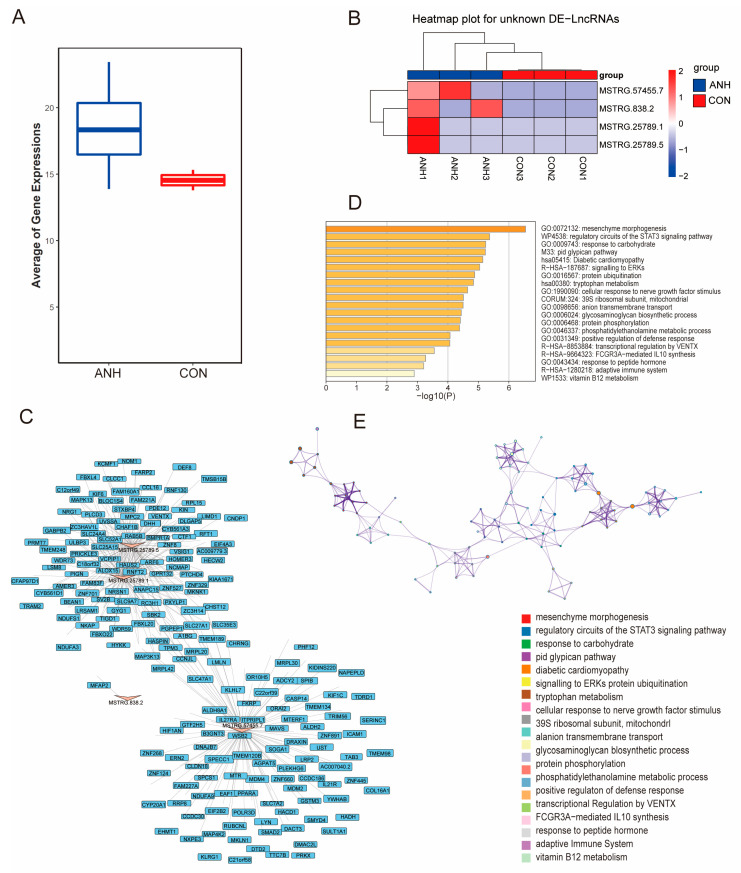
Expression and network analysis of the novel DE-lncRNAs and their corresponding targeted genes. (**A**) Boxplot showing the average expression of novel DE-lncRNAs between the ANH and control groups. (**B**) Heatmap showing the novel DE-lncRNAs between the ANH and control groups. (**C**) PPI network analysis of the four novel DE-lncRNA-targeted genes. (**D**) The top 20 Metascape-enriched KEGG pathways of the novel DE-lncRNA-targeted genes. (**E**) The top 20 Metascape PPI network analyses of novel DE-lncRNAs. ANH: ANH samples; CON: control samples.

For differential expression analysis, novel DE-lncRNAs were identified in terms of FPKM. The average expression level was much greater in the ANH group than in the control group (Figure 6A). Four significantly upregulated novel lncRNAs (MSTRG.25789.1, MSTRG.25789.5, MSTRG.57455.7, and MSTRG.838.2) were discovered in ANHs (Figure 6B). A total of 188 trans- and 2 cis-interaction target genes were obtained (Figure 6C). Metascape enrichment analysis revealed that several potential target genes were involved in pathways such as mesenchyme morphogenesis (BMPR1A, SMAD2, NRG1, MDM2, MDM4, LRP2, ALDH2, PPARA, PRKX, EHMT1, ANAPC15, FBXO22, and STXBP4), regulatory circuits of the STAT3 signaling pathway (IL27RA, IL21R, MAPK13, ALOX15, RC3H1, MAVs, SLC27A1, ICAM1, and CTF1), and cellular response to nerve growth factor stimulus (ARF6, EIF4A3, and KIDINS220) (Figure 6D,E). The specific roles of these novel DE-lncRNAs remain to be studied further.

## 4. Discussion

ANH represents a wide range of urinary diseases, but not all instances of ANH indicate an underlying pathology. Transient hydronephrosis does not correlate with postpartum pathology during pregnancy. However, stable, persistent, or worsening ANH varies and needs further investigation. Exploring biomarkers aims not only to complement the first-line US but also to confirm pathological ANH at an earlier time. In this study, children with a final diagnosis of UPJO were recruited. Two of ten labor induction procedures were used in late pregnancy because of the deteriorated ANH, as assessed by advanced MRI and US. The multidisciplinary team (MDT) evaluated the high risk of UPJO and the need for multiple operations after birth. We considered that transcriptional changes might vary based on timing and location. We also strictly controlled the ANH grades of the recruited samples to minimize bias related to gestational age and gender. In the discovery cohort, three cases of bilateral severe ANH were selected for transcriptome sequencing to reduce within-group differences. The cluster of mRNAs and lncRNAs in PCA indicated high similarity in each group, and the differences among groups were evident. Despite the small sample size, a rigorous sample analysis and transcriptome quality control were sufficient to ensure the reliability of the results. In the validation cohort, considering the unilateral cases would more accurately reflect the amniotic fluid of the functioning kidney, the unilateral (*n* = 4) and bilateral (*n* = 3) ANH results were also compared; there was no statistical difference in the expression levels of the three DE-lncRNAs. Although the sample size was limited, this finding suggests that DE-lncRNAs could serve as promising diagnostic markers for UPJO. More samples with prenatal ANH but no obstruction after birth will be collected to assess DE-lncRNAs as a potential predictor of UPJO in the future. We also plan to cooperate with pediatric nephrology for urine collection from UPJO. Urine sEVs-derived lncRNAs may be sequenced, or the DE-lncRNAs would be tested again postnatally to explore the possibility of sEVs-derived lncRNAs as risk factors for CKD.

AF has been used as a promising biomarker exploration for a long time. Klein et al. conducted a prospective multicenter peptidome analysis, and 98 peptides in AF, especially thymosin-β4, were identified to diagnose prenatally detected fetuses with CAKUT [30]. In clinical practice, AF cells are routinely used for fetal genetic testing in prenatal diagnosis, while the leftover supernatant is usually discarded. Therefore, it is beneficial to utilize the overlooked AF supernatant. This method allows for sample collection from the expectant mother without additional invasive procedures and considers the AF supernatant, mainly derived from fetal urine, as an excellent candidate for studying ANH. During the identification of sEVs, we purposely selected CD24 as one of markers. Keller et al. demonstrated that CD24 is secreted from exosomes into the urine and AF. Moreover, CD24 EVs found in the amniotic fluid of pregnant women originate from the fetal kidneys [28]. The sEVs extracted in this study were positive for CD24. Therefore, AF-derived sEVs contain inevitable biomarkers related to renal development that can be used for the diagnosis of fetal kidney disease. In a previous study, we described the proteomic profile of AF and confirmed that upregulated Moesin in sEVs could serve as a diagnostic marker for predicting postnatal renal obstruction in ANH patients [31]. In this study, we focused on sEV-derived noncoding RNAs in ANH.

Among the known lncRNAs, six DE-lncRNAs were identified. Four upregulated lncRNAs were selected for qRT–PCR analysis, three of which exhibited significant differences between the ANH and control samples. The results not only verified the sequencing data but also explored the potential of these genes as biomarkers for ANH. In particular, ENST00000454380 confirmed its official name, LINC02863 from NCBI data; it is characterized by high expression in cells and low expression in exosomes. The biological function and mechanism of LINC02863 have seldom been reported. A high expression level of LINC02863 was also found to be a necroptosis-related lncRNA that predicts the prognosis of bladder cancer [32].

LncRNAs are widely expressed in the human body and play crucial roles in both physiological and pathological processes. The mechanism of lncRNAs regulating gene expressions is complicated. LncRNAs interact with DNA, RNA, and proteins to regulate chromatin structure, functioning, and gene transcriptional activity, while also influencing RNA splicing, stability, and translation. Additionally, lncRNAs also play a role in regulating intranuclear structure [33,34]. Pathologic ANH involves renal dysplasia or urinary tract obstruction. The obstruction leads to a series of changes including renal dilation, impaired renal function, fibrosis, and inflammatory response. Specific lncRNAs may be upregulated during these processes, and they play roles in the expression of genes related to kidney development and in regulating signaling pathways related to kidney function [35]. In the present study, the expression of LINC02863 in AF-derived sEVs was significantly increased in ANH patients. When predicting its target genes, LINC02863 was found to interact with the Paired Box 8 (PAX8) gene. PAX8 is one of nine transcription factors within the PAX gene family that may determine renal fate specification and morphogenesis [36]. PAX8 is also one of the earliest specific markers of the renal lineage and is a crucial player in kidney and urinary tract development in the urogenital system [29]. Although PAX8 is essential in early embryonic development, the study of Pax8 knockout mice showed no renal abnormalities in vivo [37,38]. However, a recent study suggested that PAX8 was essential for mesenchymal–epithelial transition (MET) in de novo nephrons during human kidney development [39]. More importantly, the renal MET downstream was initiated by PAX8 through Wnt/β-catenin signaling; it coincided with the enriched Wnt pathway by GSEA in our study. The sEV-derived LINC02863 may inhibit the target gene PAX8 through underlying mechanisms including transcription factor replacement, miRNA isolation, or mRNA instability. The inhibited PAX8 downregulates Wnt/β-catenin signaling, ultimately specifying the fate of the nephron; further mechanistic studies are needed to determine the specific mechanisms involved.

The tissue and conditional specific expression of lncRNAs suggest their potential as diagnostic biomarkers. In addition, as therapeutic targets, lncRNAs exhibit many advantages such as low potential toxicity, lack of translations, and rapid turnaround and contribute to highly effective and low-dose treatments [33]. Current therapeutic methods targeting lncRNAs include developing antibodies and small interfering RNAs (siRNAs) [40], particularly antisense oligonucleotides (ASOs) [41], aimed at inhibiting their expressions, ultimately reversing the disease process. Knocking out or modifying specific lncRNAs is also conducted to evaluate their roles in the diseases [42]. Increasingly, studies revealed that lncRNAs might be involved in the pathophysiology of kidney disorders, and new treatments that target lncRNAs to reduce kidney damage are being studied extensively [43]. However, due to the difficulty of analyses regarding RNA expression changes in fetal-related diseases, there was rare research on lncRNA treatments for fetal renal abnormalities. Our study is the first time anyone systematically analyzed global RNA expressions in ANH-derived EVs. DE-RNAs in severe ANH were probably related to fetal renal development; meanwhile, DE-lncRNAs were associated with postnatal UPJO outcomes. This study provides a foundation for further research on lncRNAs as therapeutic targets in ANH and helps clinicians as well as molecular biologists chart a clear path towards therapeutics.

Novel lncRNAs were also explored in our study. We assembled 17355 novel lncRNAs and identified four upregulated novel DE-lncRNAs that may play a role in ANH. The identification of their target genes suggested that the DE-lncRNAs were closely correlated with protein-coding genes related to mesenchymal morphogenesis and the STAT3 signaling pathway. The concerted morphogenesis of the epithelium and the development of the mesenchyme play important roles in the development of various organs. Nephrons are formed by a reciprocal transition from the mesenchyme to the epithelium in the metanephric mesenchyme [44]. It was also reported that the STAT3/mTOR pathway might be involved in the process of kidney injury [45]. The potential targets involved in these pathways are frequently linked to renal fibrosis. Among the mesenchyme morphogenesis-related genes, bone morphogenetic protein receptor type 1A (BMPR1A) and its ligand bone morphogenetic protein (BMP) play a role in renal fibrosis in chronic kidney disease [46]. Smad2 protects against TGF-beta/Smad3-mediated renal fibrosis [47]. Neuregulin-1 (NRG1) administration prevents hypertrophy and fibrosis of the cardiac and renal systems caused by angiotensin II (ANG II) infusion and endothelial NO synthase (eNOS) deficiency in a mouse model [48]. In the STAT3 signaling pathway, the inhibition of STAT3 in tubular epithelial cells prevents kidney fibrosis and nephropathy in streptozotocin (STZ)-induced diabetic mice [49]. Therefore, target genes assigned to these pathways can be crucial proteins in renal development, and sEV-derived DE-lncRNAs may be potential targets as well as biomarkers for ANH.

## 5. Conclusions

In conclusion, this study identified the characteristics of the AF-derived sEV RNA transcriptome of ANHs for the first time. Both DE-mRNAs and DE-lncRNAs were shown to be associated with abnormal fetal kidney development. We shed light on the potential of known DE-lncRNAs to serve as biomarkers and pathogenic factors in ANH, and novel lncRNAs were also predicted and functionally analyzed. According to our findings, sEV lncRNAs may play important roles in ANH and facilitate the development of lncRNA-related biomarkers or therapeutic targets.

## Figures and Tables

**Table 1:** The basic information and US results.

Patient		ANH-1	ANH-2	ANH-3	Con-1	Con-2	Con-3
Age		30	36	28	40	32	35
Amniocentesis week		24^+^	24^+^	23^+^	23^+^	19^+^	20^+^
Karyotype & CNV		46, XY	46, XY	46, XX	46, XY	46, XY	46, XX
Unilateral/Bilateral ANH		Bilateral	Bilateral	Bilateral			
US Second trimester	Grading system (grade)	^1^ L: III; ^2^ R: II	L: III–IV; R: II	L: III; R: II			
	APD (mm)	L: 15.8; R: 11	L: 34; R: 18	L: 31; R: 26			
	Renal pelvis splitting	√	√	√			
	Distention of the renal pelvis and calyces	√	√	√			
	Ureteral obstruction/dilatation	Obstruction (L)	Obstruction (L) Dilatation (L)	Obstruction (L)			
	Parenchymal thinning (mm)	L: 3; R:4.1	L: 2; R: 4.6	L: 3.5; R: 5			
US Third trimester	Grading system (grade)	L: IV; R: II	L: IV; R: II	L: IV; R: II			
	APD (mm)	L: 25; R: 20	L: 25.1; R: 8.6	L: 58; R: 11			
	Ureteral obstruction/dilatation	Obstruction (L) Dilatation (R)	Obstruction (L)	Obstruction (L)			
	Parenchymal thinning (mm)	L: 3; R: 4.4	L: 2.6; R: 7.8	L: 1.6; R: 6.6			
RPA (Renal pelvis aspiration) (times: volume (mL))		1st (L): 3	1st (L): 15	1st (L): 10 2nd (L): 52 3rd (L): 70 4th (L): 110			
Diagnosis after birth		UPJO	UPJO (operation: nephron-ureterectomy	UPJO (operation: right nephron-ureterectomy)			
Evaluation renal function	Average sodium (mmol)	47.6	45.1	49.8			
	Average creatinine (µmol/L)	182.8	164.9	135			

^1^ L = left kidney; ^2^ R = right kidney.

**Table 2:** The detailed information of the known DE-lncRNAs in CON group vs. ANH group.

ID	Symbol	logFC	t	*p* Value	Regulated in ANH
ENST00000521945	SFTA3	−1.83448	−5.43091	0.004118	up
ENST00000454380	AC116366.2	−1.34567	−6.9862	0.001489	up
ENST00000439928	SPATA13	−1.01094	−3.05002	0.0332	up
ENST00000455153	CDR1	−1.60484	−3.20392	0.028291	up
ENST00000523568	AC105052.3	−1.48765	−2.67951	0.049575	up
ENST00000508166	GBA3	1.48944	3.500571	0.021009	down

## Data Availability

The datasets presented in this study can be found in online repositories. The raw transcriptomic data are available at https://www.ncbi.nlm.nih.gov/bioproject/PRJNA838478, accessed on 8 February 2023.

## Supplementary Materials

The following supporting information can be downloaded at https://www.mdpi.com/article/10.3390/biomedicines13030668/s1, Figure S1: Expression analysis and validation of the selected known DE-lncRNAs. The qRT–PCR analysis of four selected sEV lncRNAs in severe ANH and control samples. ANH: ANH samples; CON: control samples. The miR-16-5p was used for normalization. The data are presented as the means ± SDs. * *p* < 0.1; ** *p* < 0.01; *** *p* < 0.001; ns indicates no significance. Table S1: The basic information and US results of the validation cohort. Table S2: The detailed clinical information of ANH patients. Table S3: Primer list for the selected four known DE-lncRNAs. Table S4: Trans-regulatory target genes for ENST00000454380.

## References

[1] Yalcinkaya F., Ozcakar Z.B. (2020). Management of antenatal hydronephrosis. Pediatr. Nephrol..

[2] Nguyen H.T., Herndon C.D., Cooper C., Gatti J., Kirsch A., Kokorowski P., Lee R., Perez-Brayfield M., Metcalfe P., Yerkes E. (2010). The Society for Fetal Urology consensus statement on the evaluation and management of antenatal hydronephrosis. J. Pediatr. Urol..

[3] Lee R.S., Cendron M., Kinnamon D.D., Nguyen H.T. (2006). Antenatal hydronephrosis as a predictor of postnatal outcome: A meta-analysis. Pediatrics.

[4] Madsen M.G. (2013). Urinary biomarkers in hydronephrosis. Dan. Med. J..

[5] Mallik M., Watson A.R. (2008). Antenatally detected urinary tract abnormalities: More detection but less action. Pediatr. Nephrol..

[6] Harding L.J., Malone P.S., Wellesley D.G. (1999). Antenatal minimal hydronephrosis: Is its follow-up an unnecessary cause of concern?. Prenat. Diagn..

[7] Chow J.S., Darge K. (2015). Multidisciplinary consensus on the classification of antenatal and postnatal urinary tract dilation (UTD classification system). Pediatr. Radiol..

[8] Merchant M.L., Rood I.M., Deegens J.K.J., Klein J.B. (2017). Isolation and characterization of urinary extracellular vesicles: Implications for biomarker discovery. Nat. Rev. Nephrol..

[9] Ergunay T., Collino F., Bianchi G., Sedrakyan S., Perin L., Bussolati B. (2024). Extracellular vesicles in kidney development and pediatric kidney diseases. Pediatr. Nephrol..

[10] Evans M.I., Andriole S., Evans S.M. (2015). Genetics: Update on prenatal screening and diagnosis. Obstet. Gynecol. Clin. N. Am..

[11] Xie J.T., Zhou Y., Gao W.Z., Li Z.Q., Xu Z., Zhou L. (2017). The relationship between amniotic fluid miRNAs and congenital obstructive nephropathy. Am. J. Transl. Res..

[12] Dixon C.L., Sheller-Miller S., Saade G.R., Fortunato S.J., Lai A., Palma C., Guanzon D., Salomon C., Menon R. (2018). Amniotic Fluid Exosome Proteomic Profile Exhibits Unique Pathways of Term and Preterm Labor. Endocrinology.

[13] Orczyk-Pawilowicz M., Jawien E., Deja S., Hirnle L., Zabek A., Mlynarz P. (2016). Metabolomics of Human Amniotic Fluid and Maternal Plasma during Normal Pregnancy. PLoS ONE.

[14] Baraldi E., Giordano G., Stocchero M., Moschino L., Zaramella P., Tran M.R., Carraro S., Romero R., Gervasi M.T. (2016). Untargeted Metabolomic Analysis of Amniotic Fluid in the Prediction of Preterm Delivery and Bronchopulmonary Dysplasia. PLoS ONE.

[15] Del Re M., Marconcini R., Pasquini G., Rofi E., Vivaldi C., Bloise F., Restante G., Arrigoni E., Caparello C., Bianco M.G. (2018). PD-L1 mRNA expression in plasma-derived exosomes is associated with response to anti-PD-1 antibodies in melanoma and NSCLC. Brit J. Cancer.

[16] Zhou R.H., Chen K.K., Zhang J.T., Xiao B.F., Huang Z.H., Ju C., Sun J., Zhang F.F., Lv X.B., Huang G.F. (2018). The decade of exosomal long RNA species: An emerging cancer antagonist. Mol. Cancer.

[17] Bhan A., Soleimani M., Mandal S.S. (2017). Long Noncoding RNA and Cancer: A New Paradigm. Cancer Res..

[18] Kopp F., Mendell J.T. (2018). Functional Classification and Experimental Dissection of Long Noncoding RNAs. Cell.

[19] Sun Z., Yang S., Zhou Q., Wang G., Song J., Li Z., Zhang Z., Xu J., Xia K., Chang Y. (2018). Emerging role of exosome-derived long non-coding RNAs in tumor microenvironment. Mol. Cancer.

[20] Wei R., Zhao L., Kong G., Liu X., Zhu S., Zhang S., Min L. (2020). Combination of Size-Exclusion Chromatography and Ultracentrifugation Improves the Proteomic Profiling of Plasma-Derived Small Extracellular Vesicles. Biol. Proced. Online.

[21] Gardiner C., Ferreira Y.J., Dragovic R.A., Redman C.W., Sargent I.L. (2013). Extracellular vesicle sizing and enumeration by nanoparticle tracking analysis. J. Extracell. Vesicles.

[22] Dragovic R.A., Gardiner C., Brooks A.S., Tannetta D.S., Ferguson D.J., Hole P., Carr B., Redman C.W., Harris A.L., Dobson P.J. (2011). Sizing and phenotyping of cellular vesicles using Nanoparticle Tracking Analysis. Nanomedicine.

[23] van der Pol E., Coumans F.A., Grootemaat A.E., Gardiner C., Sargent I.L., Harrison P., Sturk A., van Leeuwen T.G., Nieuwland R. (2014). Particle size distribution of exosomes and microvesicles determined by transmission electron microscopy, flow cytometry, nanoparticle tracking analysis, and resistive pulse sensing. J. Thromb. Haemost..

[24] Kim D., Langmead B., Salzberg S.L. (2015). HISAT: A fast spliced aligner with low memory requirements. Nat. Methods.

[25] Pertea M., Pertea G.M., Antonescu C.M., Chang T.C., Mendell J.T., Salzberg S.L. (2015). StringTie enables improved reconstruction of a transcriptome from RNA-seq reads. Nat. Biotechnol..

[26] Love M.I., Huber W., Anders S. (2014). Moderated estimation of fold change and dispersion for RNA-seq data with DESeq2. Genome Biol..

[27] Welsh J.A., Goberdhan D.C.I., O’Driscoll L., Buzas E.I., Blenkiron C., Bussolati B., Cai H., Di Vizio D., Driedonks T.A.P., Erdbrugger U. (2024). Minimal information for studies of extracellular vesicles (MISEV2023): From basic to advanced approaches. J. Extracell. Vesicles.

[28] Keller S., Rupp C., Stoeck A., Runz S., Fogel M., Lugert S., Hager H.D., Abdel-Bakky M.S., Gutwein P., Altevogt P. (2007). CD24 is a marker of exosomes secreted into urine and amniotic fluid. Kidney Int..

[29] Sharma R., Sanchez-Ferras O., Bouchard M. (2015). Pax genes in renal development, disease and regeneration. Semin. Cell Dev. Biol..

[30] Klein J., Buffin-Meyer B., Boizard F., Moussaoui N., Lescat O., Breuil B., Fedou C., Feuillet G., Casemayou A., Neau E. (2021). Amniotic fluid peptides predict postnatal kidney survival in developmental kidney disease. Kidney Int..

[31] Li J., Fu Y., Liu Q., Shen K., Yao R., Fu Y., Lu Y., Xie M., Jian W., Guo M. (2023). Multiomics-based study of amniotic fluid small extracellular vesicles identified Moesin as a biomarker for antenatal hydronephrosis. Clin. Transl. Med..

[32] Jin Y., Li J., Tang C., He K., Shan D., Yan S., Deng G. (2023). A risk signature of necroptosis-related lncRNA to predict prognosis and probe molecular characteristics for male with bladder cancer. Medicine.

[33] Statello L., Guo C.J., Chen L.L., Huarte M. (2021). Gene regulation by long non-coding RNAs and its biological functions. Nat. Rev. Mol. Cell Biol..

[34] Mattick J.S., Amaral P.P., Carninci P., Carpenter S., Chang H.Y., Chen L.L., Chen R., Dean C., Dinger M.E., Fitzgerald K.A. (2023). Long non-coding RNAs: Definitions, functions, challenges and recommendations. Nat. Rev. Mol. Cell Biol..

[35] Dong X., Cao R., Li Q., Yin L. (2022). The Long Noncoding RNA-H19 Mediates the Progression of Fibrosis from Acute Kidney Injury to Chronic Kidney Disease by Regulating the miR-196a/Wnt/beta-Catenin Signaling. Nephron.

[36] Bouchard M., Souabni A., Mandler M., Neubüser A., Busslinger M. (2002). Nephric lineage specification by Pax2 and Pax8. Gene Dev..

[37] Mansouri A., Chowdhury K., Gruss P. (1998). Follicular cells of the thyroid gland require Pax8 gene function. Nat. Genet..

[38] Kakun R.R., Melamed Z., Perets R. (2022). PAX8 in the Junction between Development and Tumorigenesis. Int. J. Mol. Sci..

[39] Ng-Blichfeldt J.P., Stewart B.J., Clatworthy M.R., Williams J.M., Roper K. (2024). Identification of a core transcriptional program driving the human renal mesenchymal-to-epithelial transition. Dev. Cell.

[40] Khorkova O., Wahlestedt C. (2017). Oligonucleotide therapies for disorders of the nervous system. Nat. Biotechnol..

[41] Lee J.S., Mendell J.T. (2020). Antisense-Mediated Transcript Knockdown Triggers Premature Transcription Termination. Mol. Cell.

[42] Qi L.S., Larson M.H., Gilbert L.A., Doudna J.A., Weissman J.S., Arkin A.P., Lim W.A. (2013). Repurposing CRISPR as an RNA-guided platform for sequence-specific control of gene expression. Cell.

[43] Moreno J.A., Hamza E., Guerrero-Hue M., Rayego-Mateos S., Garcia-Caballero C., Vallejo-Mudarra M., Metzinger L., Metzinger-Le Meuth V. (2021). Non-Coding RNAs in Kidney Diseases: The Long and Short of Them. Int. J. Mol. Sci..

[44] San Agustin J.T., Klena N., Granath K., Panigrahy A., Stewart E., Devine W., Strittmatter L., Jonassen J.A., Liu X., Lo C.W. (2016). Genetic link between renal birth defects and congenital heart disease. Nat. Commun..

[45] Zheng S., Liu J., Zhao Z., Song R. (2020). Role of STAT3/mTOR pathway in chronic kidney injury. Am. J. Transl. Res..

[46] Vigolo E., Marko L., Hinze C., Muller D.N., Schmidt-Ullrich R., Schmidt-Ott K.M. (2019). Canonical BMP signaling in tubular cells mediates recovery after acute kidney injury. Kidney Int..

[47] Lan H.Y. (2011). Diverse roles of TGF-beta/Smads in renal fibrosis and inflammation. Int. J. Biol. Sci..

[48] Shakeri H., Boen J.R.A., De Moudt S., Hendrickx J.O., Leloup A.J.A., Jacobs G., De Meyer G.R.Y., De Keulenaer G.W., Guns P.D.F., Segers V.F.M. (2021). Neuregulin-1 compensates for endothelial nitric oxide synthase deficiency. Am. J. Physiol. Heart Circ. Physiol..

[49] Zheng C., Huang L., Luo W., Yu W., Hu X., Guan X., Cai Y., Zou C., Yin H., Xu Z. (2019). Inhibition of STAT3 in tubular epithelial cells prevents kidney fibrosis and nephropathy in STZ-induced diabetic mice. Cell Death Dis..

