# Supplementary material for: Development of LncRNA Biomarkers in Extracellular Vesicle of Amniotic Fluid Associated with Antenatal Hydronephrosis
Source: Biomedicines. 2025 Mar 8;13(3):668. doi: 10.3390/biomedicines13030668 (PMC11940114; doi:10.3390/biomedicines13030668)
Supplement: Supplementary file 1 [file biomedicines-13-00668-s001.zip › Table S1.pdf]

Supplementary Table S1 The basic information and US results of the validation cohort.

**Patient**		**ANH-4**	**ANH-5**	**ANH-6**	**ANH-7**	**ANH-8**	**ANH-9**	**ANH-10**
**Age**		28	27	35	31	27	31	30
**Amniocentesis week**		21^{+}	24^{+}	18^{+}	23^{+}	25^{+}	22^{+}	24^{+}
**Karyotype&CNV**		46, XX	46, XY	46, XX	46, XY	46, XY	46, XY	46, XX
**Unilateral/Bilateral ANH**		Left	Bilateral	Left	Left	Bilateral	Bilateral	Left
**US** Second trimester	**Grading system (grade)**	III	IV	IV	IV	IV	III	III
	**APD (mm)**	14	19	16	7.8	16	13	11
	**Renal pelvis splitting**	√	√	√	√	√	√	√
	**Distention of the renal pelvis and calyces**	√	√	√	√	√	√	√
	**Ureteral obstruction/dilatation**	Both	Obstruction	Both	Both	Both	Obstruction	Obstruction
	**Parenchymal thinning(mm)**	3	2.9	1.5	1.6	2	4.1	4.5
**US** Third trimester	**Grading system (grade)**	IV	IV	\	IV	IV	IV	IV
	**APD (mm)**	18	19	\	17.6	19	16	15
	**Ureteral obstruction/dilatation**	Both	Obstruction	\	Both	Both	Obstruction	Obstruction
	**Parenchymal thinning(mm)**	2.3	2.9	\	1.5	1.7	3.9	6
**Diagnosis after birth**		UPJO	UPJO(operation: Double-J Ureteral Stent Insertion	odinopoeia	UPJO(operation: left nephron-ureterectomy)	odinopoeia	UPJO	UPJO
