# Supplementary material for: Development of LncRNA Biomarkers in Extracellular Vesicle of Amniotic Fluid Associated with Antenatal Hydronephrosis
Source: Biomedicines. 2025 Mar 8;13(3):668. doi: 10.3390/biomedicines13030668 (PMC11940114; doi:10.3390/biomedicines13030668)
Supplement: Supplementary file 1 [file biomedicines-13-00668-s001.zip › Table S2.pdf]

Supplementary Table S2 The detailed clinical information of ANH patients

Sample	Age	Amniocentesis week	Blood pressure	Mode of pregnancy	Maternal health conditions	Medications
ANH-1	30	24+	106/62mmHg	Natural conception	1.The patient experienced no symptoms such as dizziness, headaches, chest tightness, palpitations, shortness of breath, abdominal pain, vaginal bleeding, or any other discomfort during pregnancy.<br>2.The patient denies any history of hypertension, heart disease, diabetes, kidney disease, blood disorders, or drug allergies.<br>3. No family history of genetic diseases.<br>4. No history of infections.<br>5 Alpha-thalassemia carrier. Spouse is normal.	1.Ferrous succinate (a form of iron supplement)<br>2.Calcium glucophosphate with vitamin D2 (a compound that supports bone health)<br>3. Lysamine-glucozinc granules (a supplement for enhancing zinc absorption)<br>4.Blood Enrichment Nutri-Gel (a supplement used for individuals with thalassemia)
ANH-2	36	24+	116/78 mmHg	Natural conception	1.The patient experienced no symptoms such as dizziness, headaches, chest tightness, palpitations, shortness of breath, abdominal pain, vaginal bleeding, or any other discomfort during pregnancy.<br>2.The patient denies any history of hypertension, heart disease, diabetes, kidney disease, blood disorders, or drug allergies.	Routine supplements without special drugs during pregnancy:<br>1.Ferrous succinate (a form of iron supplement)<br>2.Calcium<br>3.Vitamin

					3. No family history of genetic diseases.<br>4. No history of infections.	
ANH-3	28	23+	120/76mmHg	Natural conception	1.The patient experienced no symptoms such as dizziness, headaches, chest tightness, palpitations, shortness of breath, abdominal pain, vaginal bleeding, or any other discomfort during pregnancy.<br>2.The patient denies any history of hypertension, heart disease, diabetes, kidney disease, blood disorders, or drug allergies.<br>3. No family history of genetic diseases.<br>4. No history of infections.	1.Ferrous succinate (a form of iron supplement)<br>2.Calcium glucophosphate with vitamin D2 (a compound that supports bone health)<br>3. Lysamine-glucozinc granules (a supplement for enhancing zinc absorption)<br>4.Ritodrine（Amniocentesis conducted at 23^{+}, tablets were prescribed after 35^{+}, no effect on the sample）
ANH-4	28	21+	129/69mmHg	Natural conception	1.The patient experienced no symptoms such as dizziness, headaches, chest tightness, palpitations, shortness of breath, abdominal pain, vaginal bleeding, or any other discomfort during pregnancy.<br>2.The patient denies any history of hypertension, heart disease, diabetes, kidney disease, blood disorders, or drug allergies.<br>3. No family history of genetic diseases.<br>4. No history of infections.	Routine supplements without special drugs during pregnancy:<br>1.Ferrous succinate (a form of iron supplement)<br>2.Calcium glucophosphate with vitamin D2 (a compound that supports bone health)<br>3. Lysamine-glucozinc granules (a supplement for enhancing zinc absorption)

ANH-5	27	24+	121/78mmHg	Natural conception	1.The patient experienced no symptoms such as dizziness, headaches, chest tightness, palpitations, shortness of breath, abdominal pain, vaginal bleeding, or any other discomfort during pregnancy.<br>2.The patient denies any history of hypertension, heart disease, diabetes, kidney disease, blood disorders, or drug allergies.<br>3. No family history of genetic diseases.<br>4. No history of infections.	Routine supplements without special drugs during pregnancy:<br>1.Ferrous succinate (a form of iron supplement)<br>2.Calcium<br>3.Vitamin
ANH-6	35	18+	113/68 mmHg	Natural conception	1.The patient experienced no symptoms such as dizziness, headaches, chest tightness, palpitations, shortness of breath, abdominal pain, vaginal bleeding, or any other discomfort during pregnancy.<br>2.The patient denies any history of hypertension, heart disease, diabetes, kidney disease, blood disorders, or drug allergies.<br>3. No family history of genetic diseases.<br>4. No history of infections.	Routine supplements without special drugs during pregnancy:<br>1.Ferrous succinate (a form of iron supplement)<br>2.Calcium<br>3.Vitamin

ANH-7	31	23+	117/70mmHg	Natural conception	1.The patient experienced no symptoms such as dizziness, headaches, chest tightness, palpitations, shortness of breath, abdominal pain, vaginal bleeding, or any other discomfort during pregnancy.<br>2.The patient denies any history of hypertension, heart disease, diabetes, kidney disease, blood disorders, or drug allergies.<br>3. No family history of genetic diseases.<br>4. No history of infections.	Routine supplements without special drugs during pregnancy:<br>1.Ferrous succinate (a form of iron supplement)<br>2.Calcium<br>3.Vitamin
ANH-8	27	25+	105/72mmHg	Natural conception	1.The patient experienced no symptoms such as dizziness, headaches, chest tightness, palpitations, shortness of breath, abdominal pain, vaginal bleeding, or any other discomfort during pregnancy.<br>2.The patient denies any history of hypertension, heart disease, diabetes, kidney disease, blood disorders, or drug allergies.<br>3. No family history of genetic diseases.<br>4. No history of infections.	Routine supplements without special drugs during pregnancy:<br>1.Ferrous succinate (a form of iron supplement)<br>2.Calcium<br>3.Vitamin

ANH-9	31	22+	96/65mmHg	IVF-ET (in vitro fertilization-embryo transfer)	1.The patient experienced no symptoms such as dizziness, headaches, chest tightness, palpitations, shortness of breath, abdominal pain, vaginal bleeding, or any other discomfort during pregnancy.<br>2.The patient denies any history of hypertension, heart disease, diabetes, kidney disease, blood disorders, or drug allergies.<br>3. No family history of genetic diseases.<br>4. No history of infections.<br>5 Alpha-thalassemia carrier. Spouse is normal.	1.Multivitamin Iron Oral Solution (a form of iron supplement)<br>2.Vitamin D Drops<br>3.Lysamine-glucozinc granules (a supplement for enhancing zinc absorption)<br>4.Dexamethasone (DXMS)<br>（Amniocentesis conducted at 23+, DXMS were injected after 35+, no effect on the sample）
ANH-10	30	24+	108/72mmHg	Natural conception	1.The patient experienced no symptoms such as dizziness, headaches, chest tightness, palpitations, shortness of breath, abdominal pain, vaginal bleeding, or any other discomfort during pregnancy.<br>2.The patient denies any history of hypertension, heart disease, diabetes, kidney disease, blood disorders, or drug allergies.<br>3. No family history of genetic diseases.<br>4. No history of infections.	Routine supplements without special drugs during pregnancy:<br>1.Polysaccharide iron complex capsules (a form of iron supplement)<br>2.Calcium glucophosphate with vitamin D2 (a compound that supports bone health)<br>3. Lysamine-glucozinc granules (a supplement for enhancing zinc absorption)<br>4.Multivitamin
