# Supplementary material for: Development of LncRNA Biomarkers in Extracellular Vesicle of Amniotic Fluid Associated with Antenatal Hydronephrosis
Source: Biomedicines. 2025 Mar 8;13(3):668. doi: 10.3390/biomedicines13030668 (PMC11940114; doi:10.3390/biomedicines13030668)
Supplement: Supplementary file 1 [file biomedicines-13-00668-s001.zip › Table S3.pdf]

Supplementary Table S3 Primer list for the selected four known DE-lncRNAs

lncRNA	Symbol	Primer Sequence (5′-3′)
ENST00000521945	SFTA3	F: GGAGTTCATCCGAGGCCAAG
		R: TGCTCAGTTGGGGGTCTTTTT
ENST00000454380	AC116366.2	F: TCTGTGAGTAAGGAGCCAGC
		R: CCTGGAAACGCAGACCATGA
ENST00000439928	SPATA13	F: CAGTCCTCAGCTTCAAGGGG
		R: AGCACACTACGCAATCTCCC
ENST00000455153	CDR1	F: TCTGCTCGTCTTCCAACATC
		R: AGATCAGCACACTGGAGACG
	β-actin	F: AGAGCTACGAGCTGCCTGAC
		R: AGCACTGTGTTGGCGTACAG
	hsa-miR-16-5p	F: AACACGCTAGCAGCACGTAAA
		R: GCAGGGTCCGAGGTATTCG
