# Supplementary material for: Development of LncRNA Biomarkers in Extracellular Vesicle of Amniotic Fluid Associated with Antenatal Hydronephrosis
Source: Biomedicines. 2025 Mar 8;13(3):668. doi: 10.3390/biomedicines13030668 (PMC11940114; doi:10.3390/biomedicines13030668)
Supplement: Supplementary file 1 [file biomedicines-13-00668-s001.zip › Table S4.pdf]

Supplementary Table S4 Trans-regulatory target genes for ENST00000454380

Query	Length_Query	Target	Length_Target	dG	ndG	Start_Position_Query	End_Position_Query	Start_Position_Target	End_Position_Target
LINC02863	767	ENSG00000086506∣ENST00000199708∣HBQ1	528	-48.02	-0.1074	1	472	57	528
LINC02863	767	ENSG00000130037∣ENST00000252321∣KCNA5	2910	-54.78	-0.1196	1	462	2449	2910
LINC02863	767	ENSG00000125618∣ENST00000263335∣PAX8	3735	-54.27	-0.1028	1	530	3206	3735
LINC02863	767	ENSG00000179029∣ENST00000316425∣TMEM107	756	-42.09	-0.1022	1	444	313	756
LINC02863	767	ENSG00000157827∣ENST00000288670∣FMNL2	5630	-49.03	-0.137	1	427	5204	5630
LINC02863	767	ENSG00000155115∣ENST00000329970∣GTF3C6	788	-29.13	-0.1004	1	305	484	788
LINC02863	767	ENSG00000163743∣ENST00000324439∣RCHY1	4408	-36.14	-0.1295	1	321	4088	4408
LINC02863	767	ENSG00000116871∣ENST00000316156∣MAP7D1	3456	-51.08	-0.1051	1	512	2945	3456
LINC02863	767	ENSG00000136156∣ENST00000378549∣ITM2B	805	-38.58	-0.1099	1	394	412	805
LINC02863	767	ENSG00000125618∣ENST00000348715∣PAX8	3966	-54.27	-0.1028	1	530	3437	3966
LINC02863	767	ENSG00000116717∣ENST00000370985∣GADD45A	902	-55.12	-0.1058	1	555	348	902
LINC02863	767	ENSG00000170160∣ENST00000360524∣CCDC144A	5830	-36.43	-0.1149	1	370	5461	5830
LINC02863	767	ENSG00000101198∣ENST000003	506	-41.54	-0.1306	1	336	171	506

		70317|NKAIN4							
LINC02863	767	ENSG00000169682|ENST00000352260|SPNS1	1948	-56.16	-0.1213	1	472	1477	1948
LINC02863	767	ENSG00000173214|ENST00000368847|MFSD4B	5632	-41.06	-0.1122	1	393	5240	5632
LINC02863	767	ENSG00000018189|ENST00000417478|RUFY3	2143	-50.12	-0.1051	1	517	1627	2143
LINC02863	767	ENSG00000179029|ENST00000431792|TMEM107	455	-31.16	-0.106	1	332	124	455
LINC02863	767	ENSG00000198286|ENST00000396946|CARD11	4287	-53.68	-0.1017	1	570	3718	4287
LINC02863	767	ENSG00000120725|ENST00000508639|SIL1	675	-55.46	-0.112	1	503	173	675
LINC02863	767	ENSG00000144218|ENST00000416492|AFF3	545	-28.35	-0.1233	1	253	293	545
LINC02863	767	ENSG00000234545|ENST00000456502|FAM133B	253	-23.44	-0.1046	30	282	1	253
LINC02863	767	ENSG00000130770|ENST00000468425|ATP5IF1	539	-29.18	-0.1727	1	176	364	539
LINC02863	767	ENSG00000183474|ENST00000380729|GTF2H2C	4555	-41.7	-0.126	1	366	4190	4555
LINC02863	767	ENSG00000213186|ENST00000494486|TRIM59	577	-29.07	-0.1378	1	229	349	577
LINC02863	767	ENSG00000187164|ENST00000392901|SHTN1	2190	-36.93	-0.1176	1	370	1821	2190
LINC02863	767	ENSG00000067445|ENST00000427099|TRO	562	-34.08	-0.1593	1	265	298	562
LINC02863	767	ENSG00000125618|ENST000004	4055	-54.27	-0.1028	1	540	3516	4055

		29538|PAX8							
LINC02863	767	ENSG00000234545|ENST00000445716|FAM133B	2367	-24.6	-0.1084	1	239	2129	2367
LINC02863	767	ENSG00000164308|ENST00000512869|ERAP2	282	-17.33	-0.1025	1	191	92	282
LINC02863	767	ENSG00000144218|ENST00000415384|AFF3	574	-46.01	-0.1679	1	282	293	574
LINC02863	767	ENSG00000144218|ENST00000427118|AFF3	528	-45.76	-0.1652	1	282	247	528
LINC02863	767	ENSG00000113758|ENST00000506117|DBN1	477	-43.54	-0.1099	1	431	47	477
LINC02863	767	ENSG00000214021|ENST00000422738|TTLL3	545	-52.02	-0.1038	27	571	1	545
LINC02863	767	ENSG00000125618|ENST00000397647|PAX8	3633	-54.27	-0.1028	1	530	3104	3633
LINC02863	767	ENSG00000120725|ENST00000505945|SIL1	250	-23.74	-0.1065	1	242	9	250
LINC02863	767	ENSG00000167978|ENST00000574340|SRRM2	579	-36.98	-0.1039	1	399	181	579
LINC02863	767	ENSG00000021461|ENST00000631161|CYP3A43	328	-30.16	-0.1113	95	422	1	328
LINC02863	767	ENSG00000106976|ENST00000636201|DNM1	100	-18.9	-0.1909	50	149	1	100
LINC02863	767	ENSG00000225691|ENST00000619148|HLA-C	147	-20.61	-0.1824	367	513	1	147
LINC02863	767	ENSG00000285390|ENST00000646781|ATP5IF1	539	-29.18	-0.1727	1	176	364	539
LINC02863	767	ENSG00000105499|ENST000005	293	-27.29	-0.1083	90	382	1	293

		96352|PLA2G4C							
LINC02863	767	ENSG00000233841|ENST00000621261|HLA-C	147	-20.28	-0.1459	46	192	1	147
LINC02863	767	ENSG00000105698|ENST00000598058|USF2	510	-47.2	-0.1017	1	465	46	510
LINC02863	767	ENSG00000166598|ENST00000681861|HSP90B1	2831	-35.69	-0.1784	1	208	2624	2831
LINC02863	767	ENSG00000198597|ENST00000706147|ZNF536	4607	-55.56	-0.1075	1	538	4070	4607
LINC02863	767	ENSG00000140995|ENST00000564836|DEF8	279	-29.14	-0.1079	292	570	1	279
LINC02863	767	ENSG00000104979|ENST00000593274|C19orf53	491	-34.56	-0.1026	1	359	133	491
LINC02863	767	ENSG00000151376|ENST00000526834|ME3	298	-37.31	-0.1408	226	523	1	298
LINC02863	767	ENSG00000140995|ENST00000567884|DEF8	572	-44.88	-0.1301	1	370	203	572
LINC02863	767	ENSG00000170160|ENST00000697416|CCDC144A	5968	-36.43	-0.1149	1	370	5599	5968
LINC02863	767	ENSG00000206452|ENST00000618255|HLA-C	147	-21.63	-0.1914	367	513	1	147
LINC02863	767	ENSG00000167978|ENST00000575009|SRRM2	1146	-37.29	-0.1133	1	349	798	1146
LINC02863	767	ENSG00000125618|ENST00000681162|PAX8	4036	-54.27	-0.1028	1	540	3497	4036
LINC02863	767	ENSG00000205923|ENST00000565480|CEMP1	519	-35.31	-0.1114	1	322	198	519
LINC02863	767	ENSG00000197712|ENST000005	1431	-38.11	-0.2049	1	221	1211	1431

		15037|FAM114A1							
LINC02863	767	ENSG00000171953|ENST00000581698|ATPAF2	471	-41.75	-0.1079	1	449	23	471
LINC02863	767	ENSG00000106244|ENST00000626285|PDAP1	120	-16.53	-0.1574	28	147	1	120
LINC02863	767	ENSG00000198795|ENST00000577461|ZNF521	338	-31.21	-0.101	310	647	1	338
LINC02863	767	ENSG00000067057|ENST00000607886|PFKP	432	-40.02	-0.1209	1	383	50	432
LINC02863	767	ENSG00000169554|ENST00000637873|ZEB2	100	-10.06	-0.1212	74	173	1	100
LINC02863	767	ENSG00000167468|ENST00000614791|GPX4	431	-45.72	-0.1089	31	461	1	431
LINC02863	767	ENSG00000140995|ENST00000561741|DEF8	580	-49.21	-0.1051	1	502	79	580
LINC02863	767	ENSG00000282665|ENST00000632053|ERICH1	100	-20.73	-0.2303	79	178	1	100
LINC02863	767	ENSG00000166598|ENST00000550595|HSP90B1	3112	-35.69	-0.1784	1	208	2905	3112
LINC02863	767	ENSG00000137496|ENST00000698841|IL18BP	244	-25.39	-0.1215	144	387	1	244
LINC02863	767	ENSG00000179008|ENST00000555476|C14orf39	482	-20.59	-0.1872	1	155	328	482
LINC02863	767	ENSG00000278615|ENST00000525675|C11orf98	405	-32.25	-0.1132	1	309	97	405
LINC02863	767	ENSG00000140995|ENST00000566079|DEF8	516	-51.39	-0.1024	61	576	1	516
LINC02863	767	ENSG00000113231|ENST000002	4606	-53.85	-0.1003	1	542	4065	4606

		64917|PDE8B							
LINC02863	767	ENSG00000117477|ENST00000367805|CCDC181	1914	-26.42	-0.1797	1	192	1723	1914
LINC02863	767	ENSG00000117477|ENST00000367806|CCDC181	1917	-26.42	-0.1797	1	192	1726	1917
LINC02863	767	ENSG00000134262|ENST00000369564|AP4B1	1225	-44.29	-0.1363	1	391	835	1225
LINC02863	767	ENSG00000178922|ENST00000372434|HYI	1025	-47.91	-0.1007	1	480	546	1025
LINC02863	767	ENSG00000198125|ENST00000419229|MB	418	-35.72	-0.1249	1	314	105	418
LINC02863	767	ENSG00000224740|ENST00000439396|FLOT1	246	-20.54	-0.1135	27	272	1	246
LINC02863	767	ENSG00000156113|ENST00000372443|KCNMA1	5059	-71.78	-0.1011	1	767	4276	5042
LINC02863	767	ENSG00000137142|ENST00000377694|IGFBPL1	3566	-57.39	-0.1055	1	597	2970	3566
LINC02863	767	ENSG00000160360|ENST00000392944|GPSM1	1111	-67.17	-0.1282	1	547	565	1111
LINC02863	767	ENSG00000236271|ENST00000426417|FLOT1	367	-31.24	-0.1005	130	496	1	367
LINC02863	767	ENSG00000184640|ENST00000586521|SEPTIN9	601	-43.85	-0.1294	1	374	228	601
LINC02863	767	ENSG00000138668|ENST00000507010|HNRNPD	2640	-48.29	-0.1071	281	767	1	487
LINC02863	767	ENSG00000228964|ENST00000640675|HLA-B	240	-25.94	-0.1163	274	513	1	240
LINC02863	767	ENSG00000206450|ENST000006	240	-26.53	-0.119	428	667	1	240

		39564|HLA-B							
LINC02863	767	ENSG00000236271|ENST00000445395|FLOT1	182	-25.03	-0.1455	25	206	1	182
LINC02863	767	ENSG00000161526|ENST00000584240|SAP30BP	834	-45.41	-0.1018	1	466	369	834
LINC02863	767	ENSG00000167674|ENST00000614903|HDGFL2	239	-26.11	-0.1145	327	565	1	239
LINC02863	767	ENSG00000072682|ENST00000453286|P4HA2	819	-43.39	-0.1021	1	523	297	819
LINC02863	767	ENSG00000196296|ENST00000564112|ATP2A1	195	-19.55	-0.1685	25	219	1	195
LINC02863	767	ENSG00000128524|ENST00000492758|ATP6V1F	711	-40.84	-0.105	1	432	280	711
LINC02863	767	ENSG00000051523|ENST00000568278|CYBA	439	-36.68	-0.1066	1	359	81	439
LINC02863	767	ENSG00000282302|ENST00000633557|SEPTIN9	238	-24.75	-0.1141	66	303	1	238
LINC02863	767	ENSG00000032444|ENST00000593924|PNPLA6	584	-48.9	-0.1267	1	390	195	584
LINC02863	767	ENSG00000104365|ENST00000520810|IKBKB	3938	-53.25	-0.1078	1	567	3372	3938
LINC02863	767	ENSG00000164093|ENST00000644743|PITX2	2294	-49.5	-0.1055	1	487	1808	2294
LINC02863	767	ENSG00000188153|ENST00000504541|COL4A5	859	-21.21	-0.1198	1	198	662	859
LINC02863	767	ENSG00000179912|ENST00000448732|R3HDM2	195	-18.1	-0.1097	2	196	1	195
LINC02863	767	ENSG00000224608|ENST000006	240	-25.94	-0.1163	274	513	1	240

		39633|HLA-B							
LINC02863	767	ENSG00000073008|ENST00000587785|PVR	356	-31.23	-0.1038	1	306	51	356
LINC02863	767	ENSG00000011243|ENST00000600247|AKAP8L	583	-32.09	-0.1009	1	353	231	583
LINC02863	767	ENSG00000166333|ENST00000627400|ILK	111	-13.03	-0.1609	10	120	1	111
LINC02863	767	ENSG00000078304|ENST00000557716|PPP2R5C	645	-23.97	-0.1115	1	283	363	645
LINC02863	767	ENSG00000149311|ENST00000639240|ATM	498	-23.82	-0.1134	1	225	274	498
LINC02863	767	ENSG00000177425|ENST00000552637|PAWR	364	-38.41	-0.1219	124	487	1	364
LINC02863	767	ENSG00000100567|ENST00000553677|PSMA3	363	-16.51	-0.1834	1	111	253	363
LINC02863	767	ENSG00000015532|ENST00000509778|XYLT2	145	-24.68	-0.1898	26	170	1	145
LINC02863	767	ENSG00000131236|ENST00000446031|CAP1	273	-29.28	-0.1109	50	322	1	273
LINC02863	767	ENSG00000280682|ENST00000627494|HYOU1	862	-47.48	-0.103	1	495	368	862
LINC02863	767	ENSG00000163806|ENST00000449210|SPDYA	758	-33	-0.1255	1	273	486	758
LINC02863	767	ENSG00000136997|ENST00000641252|MYC	345	-30.23	-0.1001	1	342	4	345
LINC02863	767	ENSG00000228964|ENST00000639999|HLA-B	147	-18.31	-0.1408	458	604	1	147
LINC02863	767	ENSG00000282302|ENST000006	104	-19.05	-0.2411	121	224	1	104

		32729|SEPTIN9							
LINC02863	767	ENSG00000103034|ENST00000567063|NDRG4	558	-48.09	-0.1028	1	491	68	558
LINC02863	767	ENSG00000232126|ENST00000639001|HLA-B	240	-26.53	-0.119	428	667	1	240
LINC02863	767	ENSG00000282302|ENST00000634088|SEPTIN9	145	-19.05	-0.2411	121	265	1	145
LINC02863	767	ENSG00000111229|ENST00000547365|ARPC3	308	-15.53	-0.1652	1	97	212	308
LINC02863	767	ENSG00000183454|ENST00000636406|GRIN2A	100	-19.03	-0.2719	52	151	1	100
LINC02863	767	ENSG00000224608|ENST00000640538|HLA-B	147	-18.31	-0.1408	458	604	1	147
LINC02863	767	ENSG00000167674|ENST00000619255|HDGFL2	320	-34.15	-0.1102	200	519	1	320
LINC02863	767	ENSG00000183454|ENST00000637188|GRIN2A	100	-17.75	-0.1909	24	123	1	100
LINC02863	767	ENSG00000166548|ENST00000564917|TK2	1156	-42.07	-0.1079	1	425	732	1156
LINC02863	767	ENSG00000223532|ENST00000640223|HLA-B	240	-25.94	-0.1163	274	513	1	240
LINC02863	767	ENSG00000278823|ENST00000632906|BMERB1	534	-28.82	-0.1237	1	299	236	534
LINC02863	767	ENSG00000156113|ENST00000640523|KCNMA1	5425	-56.55	-0.1096	1	530	4896	5425
LINC02863	767	ENSG00000102804|ENST00000472477|TSC22D1	349	-31.37	-0.1082	1	325	25	349
LINC02863	767	ENSG00000138668|ENST000005	583	-43.16	-0.1353	1	329	255	583

		09107|HNRNPD							
LINC02863	767	ENSG00000205084|ENST00000615437|TMEM231	1003	-55.61	-0.1067	1	558	446	1003
LINC02863	767	ENSG00000072958|ENST00000589822|AP1M1	562	-43.3	-0.1206	1	367	196	562
LINC02863	767	ENSG00000166780|ENST00000561692|BMERB1	534	-28.82	-0.1237	1	299	236	534
LINC02863	767	ENSG00000143702|ENST00000522191|CEP170	561	-30.56	-0.1029	1	310	252	561
LINC02863	767	ENSG00000158201|ENST00000580477|ABHD3	291	-13.03	-0.1265	1	142	150	291
LINC02863	767	ENSG00000166548|ENST00000678314|TK2	1342	-35.89	-0.1502	1	256	1087	1342
LINC02863	767	ENSG00000032444|ENST00000646984|PNPLA6	672	-47.54	-0.1068	1	529	144	672
LINC02863	767	ENSG00000166548|ENST00000677739|TK2	128	-15.08	-0.1216	69	196	1	128
LINC02863	767	ENSG00000100503|ENST00000674061|NIN	342	-23.79	-0.1425	1	194	149	342
LINC02863	767	ENSG00000166548|ENST00000677412|TK2	182	-16.57	-0.1135	1	169	14	182
LINC02863	767	ENSG00000051523|ENST00000696163|CYBA	670	-56.93	-0.1039	1	565	106	670
LINC02863	767	ENSG00000134250|ENST00000652737|NOTCH2	367	-41.54	-0.1427	38	404	1	367
LINC02863	767	ENSG00000166548|ENST00000679154|TK2	122	-17.1	-0.1943	65	186	1	122
LINC02863	767	ENSG00000127022|ENST000006	5427	-52.82	-0.1046	1	517	4911	5427

		81712|CANX							
LINC02863	767	ENSG00000166548|ENST00000676904|TK2	170	-14.83	-0.1002	10	179	1	170
LINC02863	767	ENSG00000072210|ENST00000176643|ALDH3A2	3703	-44.82	-0.1057	1	496	3208	3703
LINC02863	767	ENSG00000073350|ENST00000167462|LLGL2	3480	-75.5	-0.1047	1	767	999	1765
LINC02863	767	ENSG00000111726|ENST00000229329|CMAS	1748	-40.93	-0.1197	1	367	1382	1748
LINC02863	767	ENSG00000197562|ENST00000248139|RAB40C	2718	-56.83	-0.1047	1	576	2143	2718
LINC02863	767	ENSG00000087338|ENST00000282570|GMCL1	4161	-52.67	-0.1317	1	421	3741	4161
LINC02863	767	ENSG00000163320|ENST00000309534|CGGBP1	4495	-41.33	-0.1065	1	389	4107	4495
LINC02863	767	ENSG00000072210|ENST00000339618|ALDH3A2	3828	-44.82	-0.1057	1	496	3333	3828
LINC02863	767	ENSG00000186716|ENST00000305877|BCR	6783	-58.89	-0.1042	1	595	6189	6783
LINC02863	767	ENSG00000173039|ENST00000308639|RELA	2681	-48.19	-0.1034	1	516	2166	2681
LINC02863	767	ENSG00000109944|ENST00000307257|JHY	1964	-40.22	-0.1431	1	291	1674	1964
LINC02863	767	ENSG00000152315|ENST00000282146|KCNK13	2289	-50.82	-0.1091	1	502	1788	2289
LINC02863	767	ENSG00000239927|ENST00000376642|RPP21	525	-28.87	-0.1102	452	767	1	316
LINC02863	767	ENSG00000006015|ENST000003	767	-56.14	-0.1239	1	477	291	767

		58607|REX1BD							
LINC02863	767	ENSG00000188243|ENST00000355801|COMMD6	486	-29.68	-0.1182	1	308	179	486
LINC02863	767	ENSG00000125534|ENST00000370179|PPDPF	827	-53.17	-0.107	1	572	256	827
LINC02863	767	ENSG00000145879|ENST00000523535|SPINK7	299	-16.03	-0.2323	1	100	200	299
LINC02863	767	ENSG00000179152|ENST00000396078|TCAIM	2178	-39.27	-0.1227	1	321	1858	2178
LINC02863	767	ENSG00000204839|ENST00000398882|MROH6	3265	-65.11	-0.1169	1	647	2619	3265
LINC02863	767	ENSG00000005882|ENST00000503614|PDK2	947	-49.5	-0.1012	1	505	443	947
LINC02863	767	ENSG00000130592|ENST00000429923|LSP1	582	-48.26	-0.102	39	620	1	582
LINC02863	767	ENSG00000176714|ENST00000394775|CCDC121	1472	-37.18	-0.1044	1	375	1098	1472
LINC02863	767	ENSG00000072210|ENST00000395575|ALDH3A2	3276	-43.02	-0.1039	1	461	2816	3276
LINC02863	767	ENSG00000173039|ENST00000526257|RELA	215	-19.71	-0.1011	114	328	1	215
LINC02863	767	ENSG00000204839|ENST00000529971|MROH6	1680	-65.11	-0.1169	1	623	1058	1680
LINC02863	767	ENSG00000233493|ENST00000444469|TMEM238	695	-61.56	-0.1093	1	576	120	695
LINC02863	767	ENSG00000130592|ENST00000421485|LSP1	682	-57.29	-0.1016	1	615	68	682
LINC02863	767	ENSG00000130592|ENST000004	417	-40.88	-0.1014	226	642	1	417

		51814|LSP1							
LINC02863	767	ENSG00000073350|ENST00000392550|LLGL2	3553	-75.5	-0.1047	1	767	1043	1809
LINC02863	767	ENSG00000180758|ENST00000465853|GPR157	198	-20.71	-0.1126	406	603	1	198
LINC02863	767	ENSG00000068078|ENST00000507588|FGFR3	336	-32.25	-0.1037	100	435	1	336
LINC02863	767	ENSG00000107902|ENST00000392757|LHPP	840	-39.74	-0.1024	1	422	419	840
LINC02863	767	ENSG00000130592|ENST00000446808|LSP1	574	-48.26	-0.102	51	624	1	574
LINC02863	767	ENSG00000235098|ENST00000454272|ANKRD65	1506	-75.12	-0.1216	1	629	878	1506
LINC02863	767	ENSG00000163812|ENST00000455235|ZDHHC3	537	-35.83	-0.1202	1	316	222	537
LINC02863	767	ENSG00000173039|ENST00000527749|RELA	771	-41.07	-0.1245	1	379	393	771
LINC02863	767	ENSG00000148671|ENST00000416348|ADIRF	229	-19.87	-0.1266	1	167	63	229
LINC02863	767	ENSG00000181135|ENST00000530574|ZNF707	525	-40.94	-0.1016	1	423	103	525
LINC02863	767	ENSG00000085433|ENST00000528747|WDR47	580	-29.19	-0.1031	1	339	242	580
LINC02863	767	ENSG00000186847|ENST00000167586|KRT14	1636	-47.23	-0.1031	1	477	1160	1636
LINC02863	767	ENSG00000205155|ENST00000222266|PSENEN	603	-35.64	-0.1114	1	384	220	603
LINC02863	767	ENSG00000120910|ENST000002	2194	-45.96	-0.1348	1	373	1822	2194

		40139|PPP3CC							
LINC02863	767	ENSG00000144034|ENST00000272424|TPRKB	664	-22.15	-0.2215	1	189	476	664
LINC02863	767	ENSG00000157911|ENST00000288774|PEX10	2905	-54.37	-0.106	1	525	2381	2905
LINC02863	767	ENSG00000109118|ENST00000268756|PHF12	2957	-52.28	-0.1046	1	513	2445	2957
LINC02863	767	ENSG00000152082|ENST00000281871|MZT2B	599	-47.94	-0.1029	1	524	76	599
LINC02863	767	ENSG00000109118|ENST00000332830|PHF12	4506	-58.3	-0.1119	1	541	3966	4506
LINC02863	767	ENSG00000061938|ENST00000333602|TNK2	5270	-77.32	-0.1251	1	628	4643	5270
LINC02863	767	ENSG00000186787|ENST00000333933|SPIN2B	1258	-37.86	-0.1049	1	405	854	1258
LINC02863	767	ENSG00000171206|ENST00000302424|TRIM8	3048	-63.67	-0.1208	1	552	2497	3048
LINC02863	767	ENSG00000115738|ENST00000331129|ID2	623	-38.1	-0.1187	1	353	271	623
LINC02863	767	ENSG00000171862|ENST00000371953|PTEN	8515	-55.43	-0.106	1	552	7964	8515
LINC02863	767	ENSG00000132821|ENST00000373461|VSTM2L	1936	-51.85	-0.1163	1	470	1467	1936
LINC02863	767	ENSG00000185436|ENST00000374418|IFNLR1	674	-36.31	-0.1183	1	314	361	674
LINC02863	767	ENSG00000084676|ENST00000348332|NCOA1	7381	-38.79	-0.1144	1	343	7039	7381
LINC02863	767	ENSG00000128268|ENST000003	5394	-58.79	-0.1166	1	565	4830	5394

		41184|MGAT3							
LINC02863	767	ENSG00000122741|ENST00000377724|DCAF10	7906	-46.67	-0.1019	1	532	7375	7906
LINC02863	767	ENSG00000148057|ENST00000376417|IDNK	511	-35.87	-0.104	1	355	157	511
LINC02863	767	ENSG00000152082|ENST00000425361|MZT2B	455	-42.73	-0.1017	25	479	1	455
LINC02863	767	ENSG00000186787|ENST00000434397|SPIN2B	1261	-37.82	-0.1048	1	407	855	1261
LINC02863	767	ENSG00000115738|ENST00000396290|ID2	1299	-38.1	-0.1187	1	353	947	1299
LINC02863	767	ENSG00000173467|ENST00000414935|AGR3	386	-16.04	-0.1069	1	203	184	386
LINC02863	767	ENSG00000168894|ENST00000414390|RNF181	327	-30.39	-0.1041	122	448	1	327
LINC02863	767	ENSG00000187017|ENST00000478323|ESPN	270	-27.39	-0.1118	60	329	1	270
LINC02863	767	ENSG00000031003|ENST00000425075|FAM13B	3098	-37.23	-0.1354	1	283	2816	3098
LINC02863	767	ENSG00000152082|ENST00000455239|MZT2B	401	-35.18	-0.1139	1	324	78	401
LINC02863	767	ENSG00000124839|ENST00000430445|RAB17	421	-33.51	-0.2069	1	190	232	421
LINC02863	767	ENSG00000061273|ENST00000417902|HDAC7	576	-44.16	-0.1004	1	479	98	576
LINC02863	767	ENSG00000106571|ENST00000428534|GLI3	1765	-39.47	-0.1002	1	424	1342	1765
LINC02863	767	ENSG00000235169|ENST000004	547	-47.06	-0.1043	1	476	72	547

		44870|SMIM1							
LINC02863	767	ENSG00000107282|ENST00000470082|APBA1	367	-21.89	-0.1531	1	168	200	367
LINC02863	767	ENSG00000061273|ENST00000433685|HDAC7	402	-36.96	-0.1038	1	375	28	402
LINC02863	767	ENSG00000133256|ENST00000465426|PDE6B	450	-47.35	-0.1104	104	553	1	450
LINC02863	767	ENSG00000186787|ENST00000460948|SPIN2B	401	-43.84	-0.1104	106	506	1	401
LINC02863	767	ENSG00000112695|ENST00000460985|COX7A2	357	-32.71	-0.1025	257	613	1	357
LINC02863	767	ENSG00000134121|ENST00000397491|CHL1	5235	-37.07	-0.1062	1	359	4877	5235
LINC02863	767	ENSG00000189409|ENST00000435358|MMP23B	750	-46.68	-0.1338	1	361	390	750
LINC02863	767	ENSG00000187017|ENST00000475479|ESPN	360	-30.9	-0.102	1	328	33	360
LINC02863	767	ENSG00000189409|ENST00000472264|MMP23B	556	-51.61	-0.1142	1	521	36	556
LINC02863	767	ENSG00000163818|ENST00000445698|LZTFL1	444	-29.81	-0.1222	1	265	180	444
LINC02863	767	ENSG00000124839|ENST00000409576|RAB17	466	-39.55	-0.1081	1	373	94	466
LINC02863	767	ENSG00000181350|ENST00000470794|LRRC75A	3249	-59.65	-0.1205	1	581	2669	3249
LINC02863	767	ENSG00000133030|ENST00000462033|MPRIP	309	-26.74	-0.125	1	240	70	309
LINC02863	767	ENSG00000132821|ENST000004	656	-51.85	-0.1163	1	455	202	656

		48944|VSTM2L							
LINC02863	767	ENSG00000235169|ENST00000452264|SMIM1	447	-39.63	-0.1287	1	363	85	447
LINC02863	767	ENSG00000115904|ENST00000402219|SOS1	8906	-62.75	-0.1211	1	534	8373	8906
LINC02863	767	ENSG00000061273|ENST00000445237|HDAC7	364	-37.41	-0.145	1	285	80	364
LINC02863	767	ENSG00000134121|ENST00000421198|CHL1	543	-24.11	-0.1663	1	158	386	543
LINC02863	767	ENSG00000031003|ENST00000420893|FAM13B	3214	-37.23	-0.1384	1	282	2933	3214
LINC02863	767	ENSG00000157911|ENST00000447513|PEX10	2835	-54.37	-0.106	1	525	2311	2835
LINC02863	767	ENSG00000181350|ENST00000409083|LRRC75A	2656	-59.65	-0.1205	1	541	2116	2656
LINC02863	767	ENSG00000175938|ENST00000566237|ORAI3	1393	-47.82	-0.1026	1	492	902	1393
LINC02863	767	ENSG00000141574|ENST00000581954|SECTM1	561	-51.16	-0.1082	1	480	82	561
LINC02863	767	ENSG00000282092|ENST00000631914|MED16	135	-13.75	-0.105	473	607	1	135
LINC02863	767	ENSG00000269858|ENST00000595051|EGLN2	477	-38.83	-0.1055	56	532	1	477
LINC02863	767	ENSG00000179604|ENST00000630622|CDC42EP4	240	-26.78	-0.119	73	312	1	240
LINC02863	767	ENSG00000213762|ENST00000600883|ZNF134	590	-37.54	-0.1251	1	326	265	590
LINC02863	767	ENSG00000141574|ENST000005	637	-42.12	-0.1157	1	394	244	637

		82563|SECTM1							
LINC02863	767	ENSG00000179859|ENST00000640240|RNF227	353	-35.6	-0.1134	68	420	1	353
LINC02863	767	ENSG00000198561|ENST00000533189|CTNND1	498	-30.45	-0.108	1	346	153	498
LINC02863	767	ENSG00000105227|ENST00000675300|PRX	247	-29.83	-0.1286	129	375	1	247
LINC02863	767	ENSG00000099849|ENST00000528736|RASSF7	473	-56.07	-0.1277	66	538	1	473
LINC02863	767	ENSG00000182621|ENST00000637422|PLCB1	122	-12.14	-0.1517	67	188	1	122
LINC02863	767	ENSG00000175334|ENST00000533166|BANF1	1135	-51.02	-0.1018	1	603	533	1135
LINC02863	767	ENSG00000189227|ENST00000557807|C15orf61	769	-32.73	-0.1052	1	363	407	769
LINC02863	767	ENSG00000071051|ENST00000522586|NCK2	490	-32.77	-0.1008	1	356	135	490
LINC02863	767	ENSG00000072163|ENST00000545738|LIMS2	1730	-54.45	-0.1033	1	573	1158	1730
LINC02863	767	ENSG00000168301|ENST00000491093|KCTD6	408	-26.2	-0.1464	1	222	187	408
LINC02863	767	ENSG00000061938|ENST00000671880|TNK2	407	-34.49	-0.1181	1	310	98	407
LINC02863	767	ENSG00000061938|ENST00000672024|TNK2	4325	-55.23	-0.1381	1	418	3908	4325
LINC02863	767	ENSG00000034152|ENST00000526076|MAP2K3	575	-36.67	-0.1054	1	387	189	575
LINC02863	767	ENSG00000166971|ENST000005	959	-46.29	-0.1205	1	392	568	959

		68596	AKTIP							
LINC02863	767	ENSG00000170035	ENST00000602479	UBE2E3	752	-42.2	-0.1055	1	426	327	752
LINC02863	767	ENSG00000197050	ENST00000590332	ZNF420	541	-31.03	-0.1175	1	316	226	541
LINC02863	767	ENSG00000034152	ENST00000627447	MAP2K3	351	-32.04	-0.1037	71	421	1	351
LINC02863	767	ENSG00000031003	ENST00000689681	FAM13B	5579	-35.54	-0.1356	1	276	5304	5579
LINC02863	767	ENSG00000283781	ENST00000639433	TNK1	253	-26.93	-0.1171	95	347	1	253
LINC02863	767	ENSG00000273936	ENST00000629209	TNFRSF14	674	-58.65	-0.1113	1	564	111	674
LINC02863	767	ENSG00000175221	ENST00000586342	MED16	635	-57.83	-0.1055	1	561	75	635
LINC02863	767	ENSG00000157873	ENST00000482602	TNFRSF14	434	-44.62	-0.1075	131	564	1	434
LINC02863	767	ENSG00000101544	ENST00000561195	ADNP2	124	-15.94	-0.1339	37	160	1	124
LINC02863	767	ENSG00000276497	ENST00000620509	APBA1	367	-21.89	-0.1531	1	168	200	367
LINC02863	767	ENSG00000189266	ENST00000579103	PNRC2	171	-20.93	-0.1386	357	527	1	171
LINC02863	767	ENSG00000171206	ENST00000643721	TRIM8	2759	-63.67	-0.1208	1	568	2192	2759
LINC02863	767	ENSG00000105227	ENST00000675484	PRX	170	-22.49	-0.1388	186	355	1	170
LINC02863	767	ENSG00000176531	ENST000006	546	-48.46	-0.1111	1	457	90	546

		01646|PHLDB3							
LINC02863	767	ENSG00000153391|ENST00000586489|INO80C	594	-33.15	-0.1033	1	365	230	594
LINC02863	767	ENSG00000065357|ENST00000546878|DGKA	556	-31.22	-0.1174	1	283	274	556
LINC02863	767	ENSG00000273859|ENST00000633903|RASSF7	473	-56.07	-0.1277	66	538	1	473
LINC02863	767	ENSG00000144118|ENST00000631312|RALB	311	-27.49	-0.1026	58	368	1	311
LINC02863	767	ENSG00000090621|ENST00000531243|PABPC4	532	-40.83	-0.115	1	393	140	532
LINC02863	767	ENSG00000105227|ENST00000675339|PRX	149	-19.24	-0.1384	317	465	1	149
LINC02863	767	ENSG00000182621|ENST00000637204|PLCB1	100	-12.97	-0.1491	447	546	1	100
LINC02863	767	ENSG00000165795|ENST00000555657|NDRG2	578	-36.11	-0.122	1	335	244	578
LINC02863	767	ENSG00000205707|ENST00000556198|ETFRF1	233	-22.24	-0.1426	1	164	70	233
LINC02863	767	ENSG00000142528|ENST00000599155|ZNF473	558	-42.42	-0.1015	1	459	100	558
LINC02863	767	ENSG00000168101|ENST00000586252|NUDT16L1	922	-55.05	-0.1082	1	558	365	922
LINC02863	767	ENSG00000166938|ENST00000530615|DIS3L	792	-37.13	-0.1064	1	366	427	792
LINC02863	767	ENSG00000174292|ENST00000576716|TNK1	253	-26.93	-0.1171	95	347	1	253
LINC02863	767	ENSG00000189409|ENST000005	799	-53.65	-0.1597	1	394	406	799

		03792|MMP23B							
LINC02863	767	ENSG00000100485|ENST00000216373|SOS2	5508	-50.95	-0.1031	1	514	4995	5508
LINC02863	767	ENSG00000135940|ENST00000258424|COX5B	690	-40.87	-0.1724	1	262	429	690
LINC02863	767	ENSG00000102921|ENST00000262384|N4BP1	7077	-48.21	-0.1246	1	448	6630	7077
LINC02863	767	ENSG00000157259|ENST00000287957|GATAD1	4571	-44.68	-0.1291	1	411	4161	4571
LINC02863	767	ENSG00000113384|ENST00000265070|GOLPH3	2678	-43.98	-0.1257	1	371	2308	2678
LINC02863	767	ENSG00000165458|ENST00000320683|INPPL1	717	-48.97	-0.1755	1	290	428	717
LINC02863	767	ENSG00000167969|ENST00000301729|ECI1	1507	-55.29	-0.1076	1	559	949	1507
LINC02863	767	ENSG00000163624|ENST00000295887|CDS1	4309	-41.47	-0.1481	1	290	4020	4309
LINC02863	767	ENSG00000137166|ENST00000307972|FOXP4	5994	-60.66	-0.1196	1	520	5475	5994
LINC02863	767	ENSG00000078295|ENST00000338316|ADCY2	6645	-51.51	-0.1002	1	533	6113	6645
LINC02863	767	ENSG00000116954|ENST00000373001|RRAGC	2681	-51.63	-0.111	1	505	2177	2681
LINC02863	767	ENSG00000116171|ENST00000371509|SCP2	1889	-38.88	-0.1242	1	330	1560	1889
LINC02863	767	ENSG00000096968|ENST00000381652|JAK2	7023	-51.52	-0.1014	1	547	6477	7023
LINC02863	767	ENSG00000025039|ENST000003	4042	-45.71	-0.1246	1	379	3664	4042

		59203|RRAGD							
LINC02863	767	ENSG00000119574|ENST00000354590|ZBTB45	2352	-51.99	-0.1017	1	512	1841	2352
LINC02863	767	ENSG00000135926|ENST00000420341|TMBIM1	361	-32.49	-0.1045	1	313	49	361
LINC02863	767	ENSG00000069020|ENST00000436277|MAST4	582	-46.08	-0.1328	1	372	211	582
LINC02863	767	ENSG00000154359|ENST00000398246|LONRF1	3618	-57.95	-0.1145	1	522	3097	3618
LINC02863	767	ENSG00000101473|ENST00000483141|ACOT8	1187	-45.86	-0.1079	1	456	732	1187
LINC02863	767	ENSG00000125991|ENST00000451605|ERGIC3	520	-42.74	-0.1037	1	466	55	520
LINC02863	767	ENSG00000025434|ENST00000457932|NR1H3	552	-47.55	-0.1495	1	349	204	552
LINC02863	767	ENSG00000137720|ENST00000528125|C11orf1	534	-31.22	-0.1306	1	258	277	534
LINC02863	767	ENSG00000173120|ENST00000398645|KDM2A	6511	-54.4	-0.1048	1	576	5936	6511
