# Supplementary figures and images for: Development of LncRNA Biomarkers in Extracellular Vesicle of Amniotic Fluid Associated with Antenatal Hydronephrosis
Source: Biomedicines. 2025 Mar 8;13(3):668. doi: 10.3390/biomedicines13030668 (PMC11940114; doi:10.3390/biomedicines13030668)

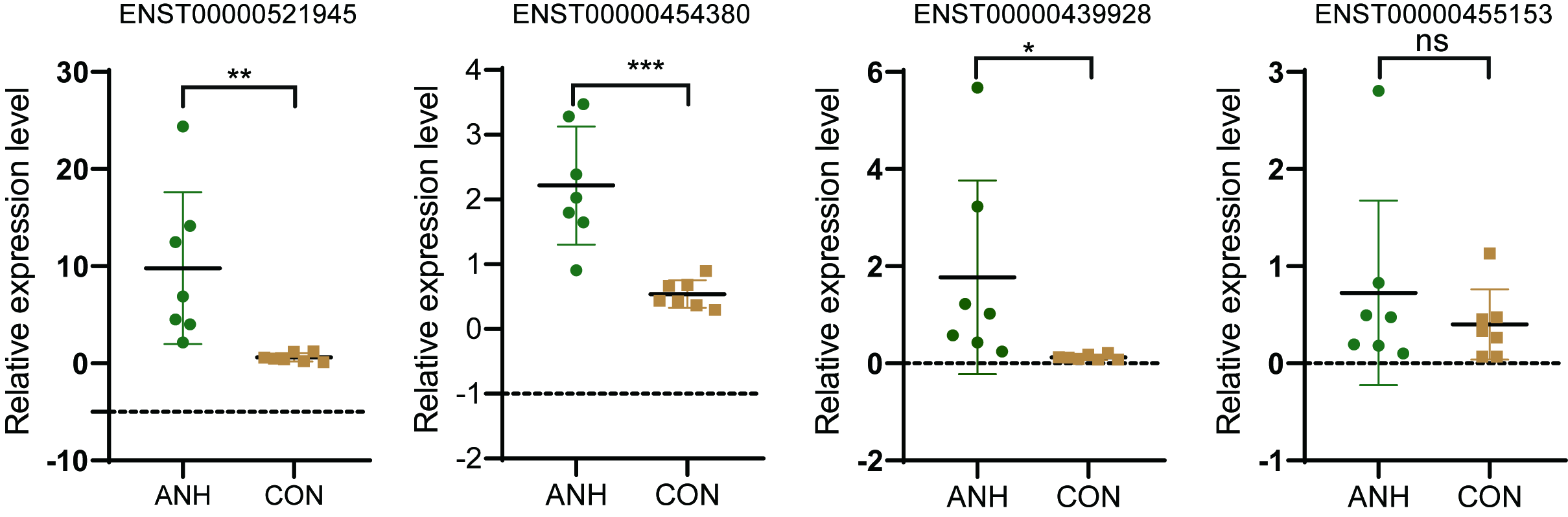

Supplement: Supplementary file 1 [file biomedicines-13-00668-s001.zip › Supplementary Figure S1.tif]
